# A Comparison of Two Spelling Brain-Computer Interfaces Based on Visual P3 and SSVEP in Locked-In Syndrome

**DOI:** 10.1371/journal.pone.0073691

**Published:** 2013-09-25

**Authors:** Adrien Combaz, Camille Chatelle, Arne Robben, Gertie Vanhoof, Ann Goeleven, Vincent Thijs, Marc M. Van Hulle, Steven Laureys

**Affiliations:** 1 Computational Neuroscience Group, Laboratory for Neuro- and Psychophysiology, KU Leuven, Leuven, Belgium; 2 Coma Science Group, Cyclotron Research Centerp, University of Liège, Liège, Belgium; 3 Department of Speech Language Pathology, ENT Head and Neck Surgery, University Hospitals Leuven, Leuven, Belgium; 4 Department of Neurology, University Hospitals Leuven, Leuven, Belgium; 5 Vesalius Research Center, VIB, Leuven, Belgium; 6 Department of Neurology, Liège University Hospital, Liège, Belgium; University Medical Center Groningen UMCG, Netherlands

## Abstract

**Objectives:**

We study the applicability of a visual P3-based and a Steady State Visually Evoked Potentials (SSVEP)-based Brain-Computer Interfaces (BCIs) for mental text spelling on a cohort of patients with incomplete Locked-In Syndrome (LIS).

**Methods:**

Seven patients performed repeated sessions with each BCI. We assessed BCI performance, mental workload and overall satisfaction for both systems. We also investigated the effect of the quality of life and level of motor impairment on the performance.

**Results:**

All seven patients were able to achieve an accuracy of 70% or more with the SSVEP-based BCI, compared to 3 patients with the P3-based BCI, showing a better performance with the SSVEP BCI than with the P3 BCI in the studied cohort. Moreover, the better performance of the SSVEP-based BCI was accompanied by a lower mental workload and a higher overall satisfaction. No relationship was found between BCI performance and level of motor impairment or quality of life.

**Conclusion:**

Our results show a better usability of the SSVEP-based BCI than the P3-based one for the sessions performed by the tested population of locked-in patients with respect to all the criteria considered. The study shows the advantage of developing alternative BCIs with respect to the traditional matrix-based P3 speller using different designs and signal modalities such as SSVEPs to build a faster, more accurate, less mentally demanding and more satisfying BCI by testing both types of BCIs on a convenience sample of LIS patients.

## Introduction

A Brain-Computer Interface (BCI) is a system aiming at establishing a non-muscular communication pathway between the brain and a computer [1]. It allows a subject to control an external device or an application using brain activity rather than muscular activity. Brain signal can be recorded, for example, via electroencephalography (EEG), which offers the advantage of being non-invasive, relatively affordable and easy to set-up. BCIs could therefore be of interest particularly for establishing a functional communication code for patients whose motor output channels are severely impaired (in most severe cases, patients with Locked-In Syndrome – LIS) typically caused by an acute ventro-pontine brainstem lesion (commonly after stroke or more rarely after traumatic brain injury; [2]), or in the end stage of amyotrophic lateral sclerosis (ALS; [3], [4]) and for whom devices that rely on residual voluntary motor activity are not effective [5].

Some of the earliest EEG-BCI systems were based on the P3 component of the Event-Related Potential (ERP; [6], [7]). The P3 is a positive deflection in the EEG time-locked to salient stimuli presented in an oddball paradigm, typically evoked over the parietal cortex, and occurs between 200 and 500 ms after stimulus onset [8]. P3-based BCIs are sometimes called ERP- or oddball-based BCI due to the fact that, although they rely mostly on the P3 component, other components (*e.g.*, occipital N1 and/or N200) may also be used for ERP detection [9], [10]. We prefer to use the more usual term P3-based BCI (no electrodes were placed over the occipital region in our study for this type of BCI).

The successful use of P3-based BCIs by a large population of healthy users was reported by [11]. Alongside, many studies have shown that this system is feasible and practical for patient groups (see *e.g.* [12]–[14], or [15] for a review). Additionally, other studies reported on the stability of the performance over time on a population of ALS patients [16]–[18].

[16] and [17] also reported on the absence of a significant correlation between the degree of impairment of the patients and their performance with the BCI system. However, [19] observed that only the performance for the two most impaired patients (out of five patients tested) was significantly lower than the performance of the control group composed of seven healthy subjects.

Other systems of interest for patients with LIS are BCIs based on Steady-State Visually Evoked Potentials (SSVEP). They rely on the psychophysiological properties of the EEG brain responses recorded from the occipital cortex during the periodic presentation of identical visual stimuli (*i.e.* flickering stimuli). When the periodic presentation is at a sufficiently high rate (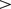
6 Hz), stable and synchronized neural oscillations at the stimulus frequency and its harmonics are evoked over the visual cortex [20]–[22]. Such BCIs are particularly attractive because SSVEPs have high signal-to-noise ratios and are less susceptible to eye movement and blink artifacts [23] as well as electromyographic artifacts [24].

Several SSVEP-based BCIs have been successfully tested with healthy subjects (see [25] for a review). To our knowledge, the only study of such system on disabled subjects was performed by [26], showing that seven healthy participants and four patients affected by muscular dystrophy at different stages were able to successfully use this system.

[27] reported on tests performed on 14 healthy participants where each of them performed one session with a P3-based BCI and another one with an SSVEP-based BCI. They observed accuracies within a similar range for both systems (77–100%), but with higher transfer rates for the SSVEP-based BCI (mean 25 bit/min) than for the P3-based one (mean 9 bit/min). They also reported that 4 subjects had too weak SSVEP responses to be exploited by the BCI and that 9 out the 10 remaining subjects preferred the SSVEP application.

To our knowledge, no study has been performed on letter-spelling BCIs in incomplete LIS (*i.e.*, quadriplegic and severely dysarthric patients showing mainly yes-no communication via eye-movements but having recovered some motor control in head or limbs; [2], [28]) comparing SSVEP- and P3-based BCI applicability. Since we are aware of the difficulty to generalize results obtained in healthy subjects to a pathological population [29], [30], we aim here at comparing a P3-based and an SSVEP-based BCIs for spelling on a cohort of patients with incomplete LIS.

In order to be suited for daily use, BCI systems need to be accurate as well as easy to use for the patient. The applicability will therefore depend not only on the achieved performance but also on the users' assessment of the mental workload associated with the task and the overall satisfaction with the system. For this reason, a comparison was conducted in terms of performance, mental workload and user satisfaction. Moreover, with regard to previous studies on P3-based BCIs, we also investigated the relationship between the observed performance, the patient's level of impairment and his/her quality of life.

## Materials and Methods

### Population

At first, eleven patients who had suffered from LIS after an acute brain insult were selected for the study. Three had to be excluded due to visual impairments or attention deficits. One patient decided to leave the study after the first BCI session on account of a severe depression. Seven patients between 21 and 61 year (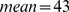, 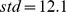, between 1 and 18 years post onset) were included for further analysis. Six had a vascular brainstem lesion and one had a traumatic brain injury (TBI). All of them were able to communicate either by eye or head movements or with a computer (see Table 1). None of them had a history of psychiatric or neurological disorders prior to the onset. No patient had a history of epilepsy. The study was approved by the ethical committees of the university hospital of the KULeuven and of the university of Liège and all participants provided informed consent. Patients were required to indicate agreement via their legal representative and via eye-coded communication (the consent procedure was also approved by the ethical committees).

**Table 1:** Clinical description and level of disability (ALSFRS-R and Patterson scales) for all patients.

Patient	Gender	Age	Etiology	Time since onset (years)	Lesions	Communication code	ALSFRS-R score	Patterson score
S1	M	21	TBI	4.5	Lesions in the right cerebellar, right frontal and left lenticular. Global atrophy, predominantly in the vermian and midbrain	Eye movements	20	2
S2	M	39	Brainstem stroke	17.5	Brainstem lesion	Eye blinks, head movement with virtual keyboard	18	2
S3	F	46	Brainstem stroke	7.7	Brainstem and right thalamic lesion	Head movement, oral communication with severe dysarthria	22	3
S4	F	49	Brainstem stroke	1.2	Brainstem lesion	Oral communication with severe dysarthria	24	3
S5	M	44	Brainstem stroke	2.9	Pontine infarcts extending into mesencephalon and right occipital lobe. Basilar artery occlusion	Head movement, oral communication with severe dysarthria	30	3
S6	M	61	Brainstem stroke	12.4	Infarct in the central part of the brainstem and infarction in the left cerebellum in the PICA territory	Eye movements, finger tapping	14	2
S7	F	41	Brainstem stroke	6.4	Recent infarcts in medulla oblongata/pons/mesencefalon. Thrombosis of basilar artery and distal right vertebral artery. Patent foramen ovale	Eye movements, head movement with virtual keyboard	17	2

For both BCI systems, all patients first performed a *preliminary session*, during which basic testings were done (see *Preliminary tests* on page 4) and the BCI settings were optimized (see *Experimental protocol* on page 6 and *Experimental protocol* on page 5). The data collected during these preliminary sessions were not considered for analysis. Additionally, all subjects performed two *acquisition sessions* (except patient S6 who could perform only 1 session with the P3-based BCI) during which they freely used the system to type words of their choice (free spelling mode). Patient S6 did not manage to use the P3 system and reported difficulties to fulfill the task; for this reason he did not want to participate in a second acquisition session with this BCI. One session lasted between one and two hours including regular breaks, and maximum effort was made to keep the patients fully concentrated; the sessions were ended when the subjects started to feel tired. The order of the sessions was randomized for each subject.

### Material

The EEG recordings were performed using a prototype of an ultra low-power 8-channel miniature EEG amplifier, which wirelessly transmits the data to an USB stick receiver at a sampling rate of 1 kHz for each channel with a resolution of 12 bits per sample. The prototype was developed by imec (see [31]) and was successfully used in several BCI studies on both healthy and disabled participants (*e.g.* [14], [32]). We used a brain-cap with large filling holes and sockets for active Ag/AgCl electrodes (ActiCap, Brain Products). The portability of the EEG equipment and the low number of required channels allowed us to easily transport the material to the patient's home and to set up the experiment in a few minutes. Although this aspect is not critical for *in-lab* experiments, with healthy subjects, it is an important characteristic of our clinical study.

For the P3 speller, the brain activity was recorded with 8 electrodes placed over the frontal, central and parietal areas of the brain, namely in positions *Fz*, *FCz*, *Cz*, *CP1*, *CP2*, *P3*, *Pz* and *P4* according to the international 10–20 system; The recording sites were the same as in [32] (see also [15], [33] for some guidelines on selecting the recording sites). For the SSVEP speller, all 8 recording electrodes were placed around the occipital part of the brain which corresponds to the visual cortex (see [34], [35]). The positions used were *P3*, *Pz*, *P4*, *PO9*, *O1*, *Oz*, *O2*, *PO10*. For both setups, the reference and ground electrodes were positioned on the left and right mastoids, respectively (*TP9*, *TP10*).

All recordings and stimulation employed MATLAB®, the stimuli were visually presented on a laptop's LCD screen (60 Hz refresh rate) and displayed and timed using the *Psychophysics Toolbox Extensions* [36], [37]. Participants sat in their chair in front of the computer screen (at about 1 meter distance).

### Preliminary tests

**Figure 1:**
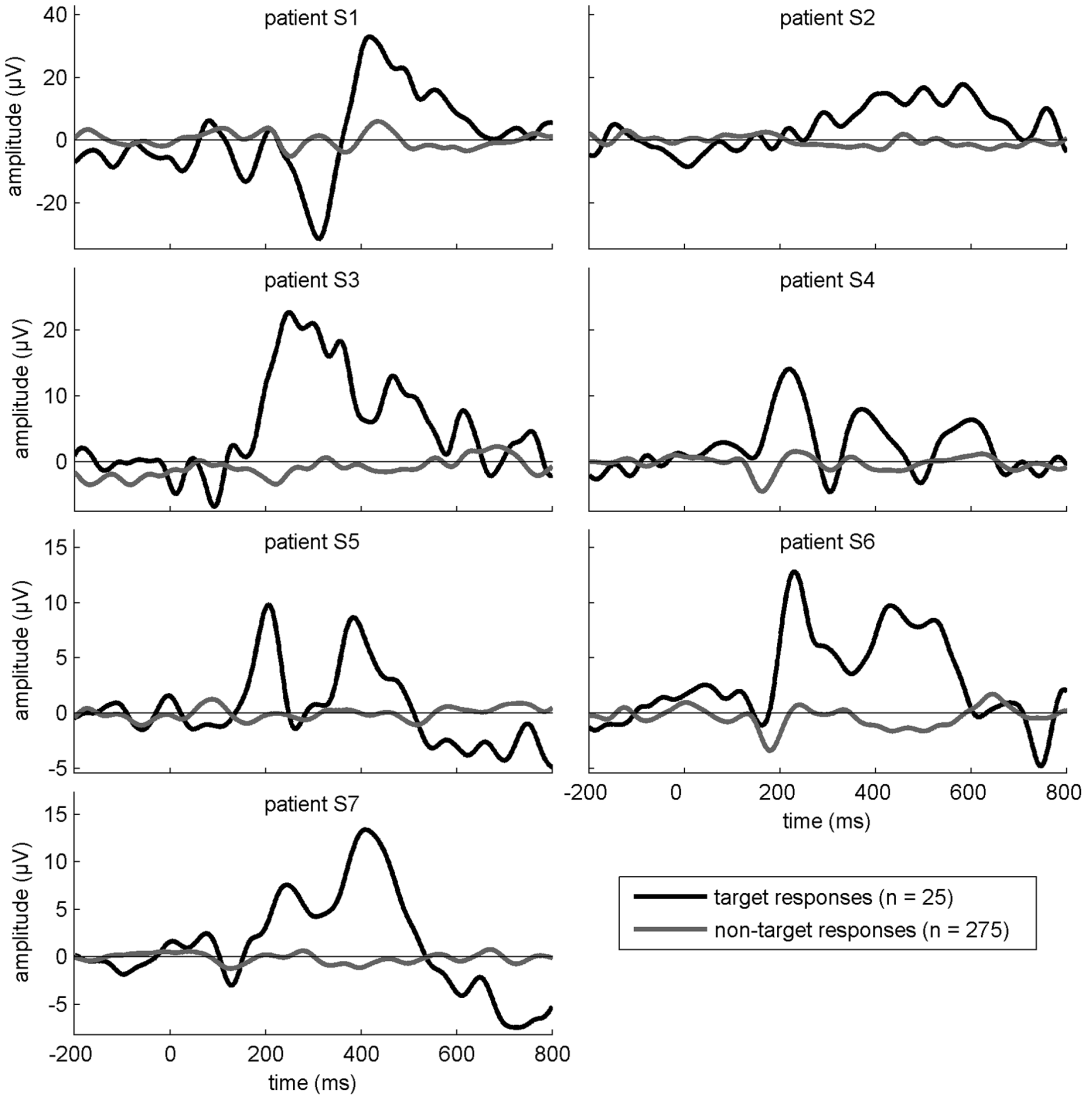
Results from the oddball experiment: average responses to target and non-target stimuli at electrode site *FCz* from 200 ms before stimulus onset until 800 ms after.

For both BCIs, we conducted a preliminary session during which initial testings were carried out by each participant. For the P3-based BCI, this test consisted of a visual oddball experiment. The patients had to attend a series of stimuli displayed consecutively and in random order on a computer screen. The stimuli consisted of 25 green crosses (*target stimuli*) and 275 red disks (*non-target stimuli*), each stimulus was displayed for 100 ms followed by a pause randomly set between 100 and 300 ms. The patients were asked to count mentally the number of green crosses appearing on the screen. This experiment allowed us to identify the P3 response of the patients and to explain them what generated this component so as to introduce them to the P3 spelling BCI. The averaged responses of each participant to target and non-target stimuli are shown in Fig. 1.

**Figure 2:**
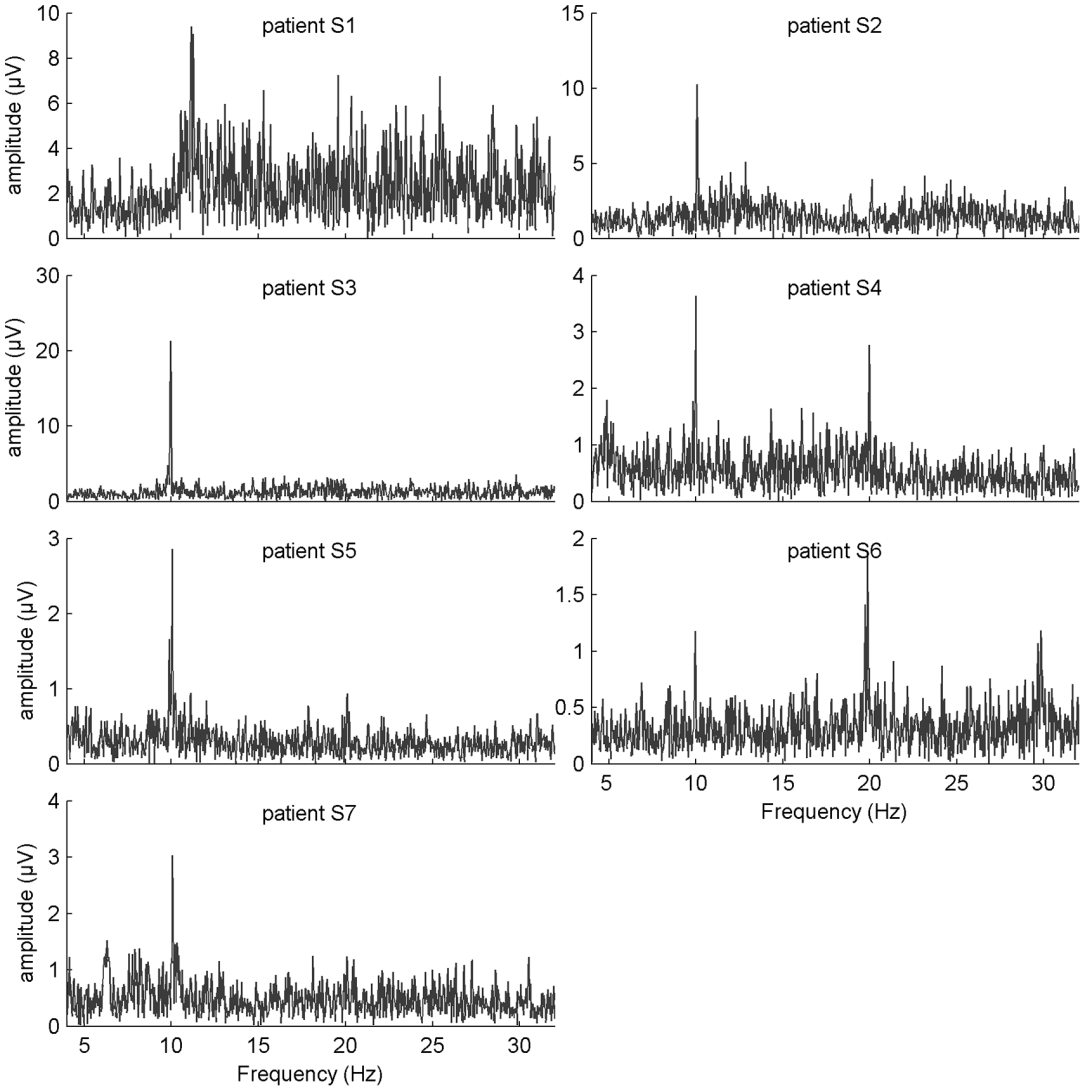
Results from the SSVEP scanning experiment: amplitude spectrum of the EEG response to the 10 Hz flickering square at electrode site *Oz* for each patient. Note that for all patients, a peak at the stimulation frequency (and sometimes at its harmonic) can be observed in the amplitude spectrum.

For the SSVEP-based BCI, the preliminary test consisted of a “*SSVEP scan*” of the participants. They were asked to focus on a red dot in the center of the screen while a white rectangle was flickering on a black background. In total 7 flickering rectangles were presented to the subjects during 20 s, each followed by a 5 s pause. In order to generate stable stimulation frequencies on the LCD screen, we used frequencies corresponding to the divisions of 60 Hz (refresh rate of the screen, see [38]) by 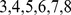 and 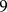 (respectively 20, 15, 12, 10, 8.57, 7.5 and 6 Hz). This experiment allowed us to introduce the patients to the SSVEP paradigm and to gain insight as to which stimulation frequencies were most appropriate. The amplitude spectrum (*Fast Fourier Transform*) of the EEG response to the 10 Hz flickering square measured at the channel *Oz* is shown for each participant in Fig. 2.

### P3 speller paradigm

#### Paradigm description

A 6 by 6 matrix of alphanumeric symbols was presented on the screen. Participants were asked to count the number of time a symbol was highlighted in order to select it.

**Figure 3:**
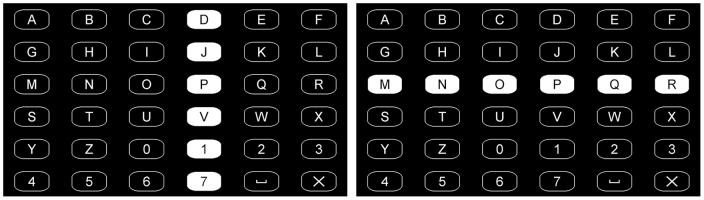
P3 BCI: row/column stimulation.

Two oddball designs were used in the study:

- The classical way of grouping symbols into stimuli using a *row/column* paradigm as shown in Fig. 3. In a 6 by 6 matrix, a *sequence of intensifications* corresponds to the flashing of each of the 6 rows and 6 columns of the matrix (in random order), the target symbol being at the intersection of the target row and target column.
- An alternative way consisting of flashing the symbols individually (*single symbol* stimulation); in that case the target stimulus corresponds to the target symbol.

It is important for the row/column paradigm to set up a pause between each stimulus so that the subject would not be too disturbed by the flashes, however the duration of each flash and pause can be adjusted for user convenience. We refer to those as *stimulus duration* and *Inter Stimulus Interval* (ISI), respectively and took values between 100 and 125 ms for both. The sum of those 2 values is referred to as the *Stimulus Onset Interval* (SOI).

The row/column paradigm has the advantage that it needs fewer stimuli for each intensification sequence (12 stimuli for the row/column *vs.* 36 for the single symbol) and thus, for a fixed number of repetitions of the flashing sequence, the communication rate is higher. While for this reason the row/column stimulation is usually preferred to the single symbol one, in some situations the latter paradigm might be more appropriate for subjects who are too disturbed by the simultaneous flashing of groups of symbols. Additionally, some factors come into play to compensate for the decreased communication rate of the single symbol paradigm such as the fact that the amplitude of the P3 is known to be inversely correlated to the probability of the target stimulus [8], [39] and the possibility with the single symbol paradigm to display the stimuli without any pause between the flashes.

This P3-based BCI with the row/column stimulation paradigm was previously tested on healthy and on stroke and ALS participants [14], [32]. Similar P3-based systems with single symbol stimulation paradigms were validated on healthy subjects [11], [40].

Although other alternatives to the traditional row/column paradigm have been suggested [11], [41]–[44], we decided to use the single symbol design because it minimizes the amount of distracting stimuli with respect to the target stimulus, which for some patients was a major concern.

#### Calibrating the system and classifying the EEG signals

As the shape of the ERP can vary greatly across subjects and sessions [45], it is necessary to tune the ERP detection algorithm prior to any spelling at each session. Hence, at the beginning of each session, we performed a calibration phase during which the participants were asked to focus consecutively on 8 symbols, randomly selected by the interface, using 10 repetitions of the sequence of intensifications. This calibration phase lasted between 3 and 6 minutes depending on the paradigm and the SOI used.

Based on the data recorded during the calibration phase, we built a classifier for the detection of the ERP. The signals were filtered between 0.3 and 15 Hz (3^rd^ order zero-phase Butterworth filter), and cut into 800 ms epochs starting from the stimuli onsets. Epochs were downsampled to 100 Hz and the data of the same classes were averaged over the desired number of trials (corresponding to the desired number of repetitions of the sequence of intensification for the spelling mode).

For each trial (stimulus), we had 640 features (8 channels ×80 data points) to classify as a response to either a target stimulus or a non-target stimulus. A linear *Support Vector Machine* (SVM; [46], [47]) with a 10-fold cross-validation and a linesearch for the optimization of the regularization parameter was built from the normalized calibration features. Training the linear SVM with the modified finite Newton method proposed by [48] took around one minute.

#### Experimental protocol

Besides the visual oddball experiment (see *Preliminary tests* on page 4), the preliminary session was also used to introduce the system, material and protocol to the patient and to optimize the BCI paradigm so as to maximize the patient's comfort with respect to the stimulus set-up. The paradigm (row/column or single symbol), as well as the stimuli duration and ISI that would be used for the remaining sessions were chosen during this preliminary session.

As mentioned in the section *Paradigm description*, to achieve a higher communication rate, preference was given to the row/column stimulation style; however when a participant reported issues in fulfilling the task or did not reach a performance above 50% with 10 repetitions of the intensification sequence and an SOI of 250 ms or higher, we turned to the single symbol paradigm. The stimulation style and parameters used for each participant are listed in Table 2 including the corresponding duration of a sequence of intensification (to obtain the stimulation time needed to communicate one symbol, this quantity has to be multiplied by the number of repetitions used) and the number of sessions in which each patient took part. Only the following sessions (acquisition sessions) were considered for data analyses.

**Table 2:** Parameters of the P3-based BCI (the number of sessions field does not include the preliminary session).

Patient	number of sessions	stimulation style	stimulus duration (ms)	Inter Stimulus Interval (ms)	flash sequence duration (s)
S1	2	single symbol	100	0	3.6
S2	2	single symbol	100	0	3.6
S3	2	row/column	100	150	3
S4	2	row/column	125	125	3
S5	2	row/column	125	125	3
S6	1	single symbol	100	50	5.4
S7	2	row/column	125	125	3

The acquisition sessions started with the calibration phase as described in the section *Calibrating the system and classifying the EEG signals*. A classifier was then built based on the calibration data and used during the rest of the session for the patients to write words of their choice (free spelling). They would first use the system with 10 repetitions of the intensification sequence. After each typed word, and depending on how much they were satisfied with their performance, the number of repetitions was increased or decreased on the patients' request, allowing them to use the system with faster or slower settings.

### SSVEP speller paradigm

#### Paradigm description

**Figure 4:**
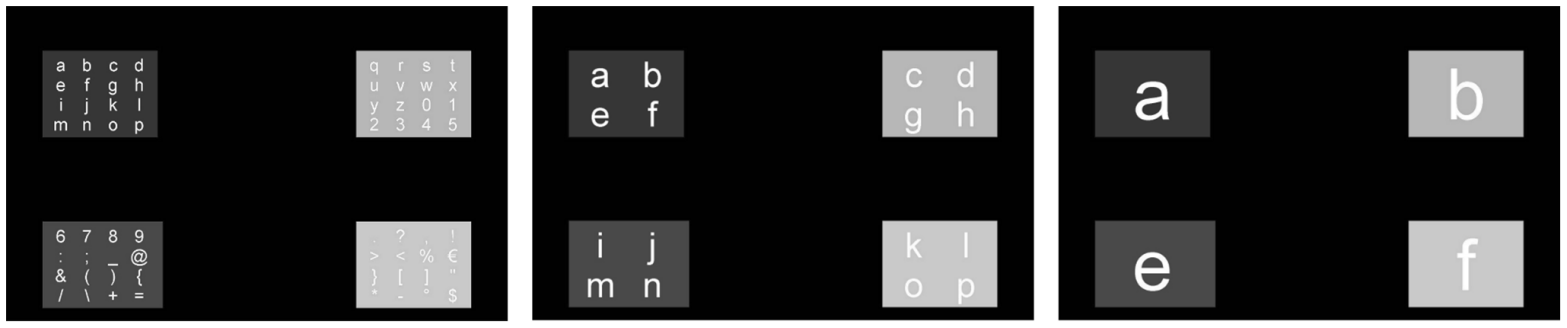
SSVEP BCI: 3 stimulation levels.

For the SSVEP speller, 64 symbols were divided into 4 quadrants (4 by 4 characters each) located in the corners of the screen (see Fig. 4). The quadrants were flickering with different frequencies, allowing the subject to select one group of characters through his/her SSVEP responses by focusing on the target quadrant (containing the target symbol). When a group of symbols was selected by the system, it was then divided in 4 new groups redistributed over the 4 quadrants so that the user can narrow down the selection. Ultimately, the target symbol was communicated after 3 successful identifications of the subject's SSVEP response. The stimulation duration for each of the 3 levels was fixed in advance in the same way as the number of intensification sequences was fixed for the P3 speller. This BCI was previously tested on healthy subjects as reported by [49].

#### Classifying the EEG signals

The signals were downsampled to 250 Hz and filtered above 4 Hz with a 4^th^ order zero-phase Butterworth filter. A notch filter was applied to remove the 50 Hz powerline interference. For the SSVEP detection we used a technique proposed by [50] (also applied by [49], [51]).

This technique consists in first applying a spatial filter to the EEG data following the *Minimum Energy Combination* method suggested by [50]. It results in a set of linear combinations of the original EEG signals for which the noise is minimized at the frequencies of interest (*i.e.* the 4 stimulation frequencies) and their harmonics.

In the second step, a scoring function was calculated for each of the 4 stimulation frequencies and the one with the highest score was identified as the target frequency. The scoring function corresponds to the average of the signal-to-noise ratio across harmonics and components of the spatially filtered signals. The signal-to-noise ratio was calculated as the ratio of the estimated signal power and the estimated noise power at the desired frequency (see [50], [51] for details).

#### Experimental protocol

As with the P3 speller, besides the SSVEP scan (see *Preliminary tests* on page 4), the preliminary session was also dedicated to familiarizing the patients with the paradigm and to choose the stimulation frequencies that would be used for the acquisition sessions. Only those later sessions were used for data analyses. The stimulation frequencies used and the number of sessions in which each subject took part are presented in Table 3.

**Table 3:** Parameters of the SSVEP-based BCI (the number of sessions field does not include the preliminary session).

Patient	number of sessions	stimulation frequencies (Hz)
S1	2	6.67–7.5–8.57–10
S2	2	7.5–8.57–10–12
S3	2	7.5–8.57–10–12
S4	2	6.67–7.5–8.57–10
S5	2	6.67–8.57–12–15
S6	2	6.67–7.5–8.57–10
S7	2	6.67–7.5–8.57–10

As this BCI does not require any calibration, patients could directly start the acquisition sessions and spell words of their choice. For each session, the participants typed their first word (s) with a stimulus duration of 10 seconds (30 seconds of stimulation per symbol). Like for the P3 speller, depending on how they were satisfied with their performance, the stimulation time was increased or decreased on the patients' request.

### Evaluation criteria

#### Measuring and comparing performance

Only the data collected during the acquisition sessions were used for analyses. When analyzing the performance of all participants for each system independently, we looked at the typing accuracy with respect to the communication rate (*i.e.* the stimulation time corresponding to the selection of one symbol). There is no clearly established accuracy threshold determining the usability of a BCI [52]. We chose to use two different thresholds. When the accuracy was below 50%, we regarded the BCI as not usable and considered it usable only when the accuracy was above 70%. The first threshold was motivated by the fact that below 50%, each symbol selected by the system would have a larger chance to be wrong than to be correct; which would seriously compromise the usability of the system [12]. The second threshold was used by [53] to evaluate their BCI system; this choice was legitimized by the work of [54] who argued that an accuracy threshold in the 70–80% range is required for a speech recognition system to be considered usable.

When comparing both systems for all patients, we have to take into account that the P3 speller offers a choice of 36 symbols, against 64 for the SSVEP speller. Indeed, for a given accuracy and speed, the system with the highest performance is the one encoding the largest amount of information, thus the one with the largest number of choices. So, if the typing accuracy and the selection speed are critical to compare the performance of our BCIs, the number of choices is also decisive in this context.

For this reason, the performance was also measured in terms of the Information Transfer Rate (ITR, see for example [55]–[58]) expressed in *bits per minute* and defined as:
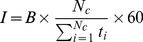
(1)where 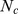 is the number of symbols communicated, 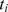 the time in seconds needed to communicate the 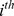 symbol and 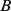 the bitrate expressed in bits per symbols and defined by:
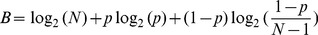
(2)where 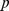 is the classification accuracy and 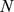 the number of possible symbols to communicate.

Like for the accuracy, we looked at the ITR values with respect to the communication rate. We also calculated for each patient a unique ITR value reflecting the performance with each system. A Wilcoxon signed-rank test (as described in [59]) was performed in order to compare the ITR values associated with each system.

#### Quality of life, satisfaction and mental workload assessment

At the end of the last session with each system, the patients were asked to assess their overall quality of life and satisfaction regarding the system used. The quality of life was assessed by means of the Anamnestic Comparative Self-Assessment scale (ACSA; [60]), whose biographical +5 and −5 scale anchors were the patients' memories of the best period in their life before LIS and their worst period ever. The satisfaction assessment was based on an analog visual scale. We asked the patient to rate his overall satisfaction with the used BCI-device between 0 indicating “not satisfied at all” and 100 meaning “absolutely satisfied”.

We used the NASA Task Load Index (TLX; [61]) to assess the mental workload associated with each system. The patients rated six domains (mental demand, physical demand, temporal demand, own performance, effort, and frustration levels) with high ratings indicating an increased workload (values were scaled between 0 and 100). Given the specific constraint of eye or head-coded communication in the surveyed patients with incomplete LIS, all the scales were administered using the “yes/no” communication code of the patient. The examiner pointed the scale until the patient said “yes” to select the desired answer. The mental workload was estimated using the Raw TLX (RTLX), defined as the average of the score given to each factor [62]–[64].

A Wilcoxon signed-rank test was performed in order to compare the mental workload associated with each system.

#### Level of impairment

The severity of motor impairment was assessed using the Patterson & Grabois scale [3], [65]. This is a 5 points scale ranging from 0 (no recovery- no return of motor function and total dependence in all activities of daily living) to 5 (no neurologic deficit).

We also used the revised ALS functional rating scale (ALSFRS-R; [66]) to characterize motor disabilities [16], [17], [58]. This scale is composed of 12 items graded from 0 (complete loss of function) to 4 (normal function) with a score range between 0 (unable to perform the tested functions) and 48 (normal functions). The 12 items explore bulbar functions (swallowing, speech and salivation), upper limb movements (handwriting and cutting food), lower limb movements (walking and climbing stairs), other mobility functions (dressing and hygiene, turning in bed) and respiration (dyspnea, orthopnea, respiratory insufficiency). Those functions are often impaired in patients suffering from LIS from other etiologies than ALS and therefore this scale is of interest to assess motor impairment in the population included in our study.

We assessed the correlation between ITR and level of impairment (from each scale) independently of the system used and separately for each BCI using respectively the Spearman partial correlation coefficient and the Spearman correlation coefficient [59]. The statistical significance was assessed via a permutation test ([67]; method 1 with n = 20000 permutations).

## Results

### Performance comparison

**Figure 5:**
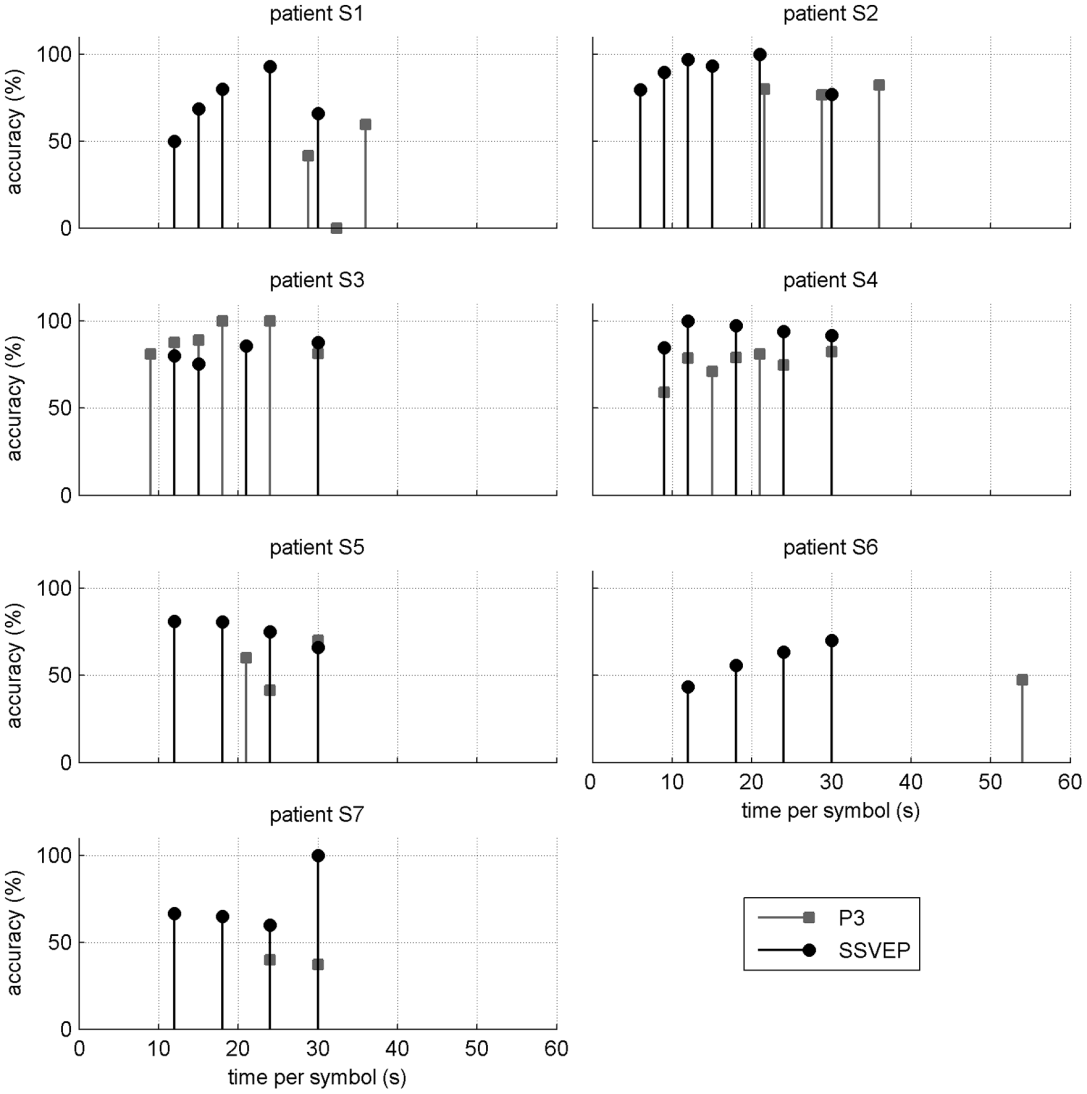
Detection accuracies for both BCIs and each patient with respect to the stimulation time per symbol. Note that for all subjects except S3, we observe higher accuracies for the SSVEP-based BCI and that for S1, S2, S5, S6 and S7 this BCI could be used with faster settings than the P3-based BCI.

The detection accuracies with respect to the stimulation time per symbol for each patient are shown in Fig. 5. More detailed information about the performances for each subject, session and BCI can be found in Supplementary Materials S1 and S2. Patient S6 did not manage to control the P3-based BCI and did not want to participate in a second session. Therefore, he did not perform the same amount of sessions with each system. For this reason, his data were excluded from all statistical analysis and average calculation, we however decided to also show his data in all results that are displayed per subject.

All patients were able to reach an accuracy above 70% with the SSVEP system; this was not the case for the P3 system as patients S1, S5, S6 and S7 failed to overcome this threshold, regardless of the settings used. Particularly, S6 and S7 did not manage to reach the 50% usability threshold with the P3 BCI. Six patients out of 7 (all except S3) performed better with the SSVEP than with the P3 system as evidenced by the higher accuracies and (except for patient S4) the faster settings that could be tried. Patient S3 achieved a slightly better performance with the P3-based BCI.

**Figure 6:**
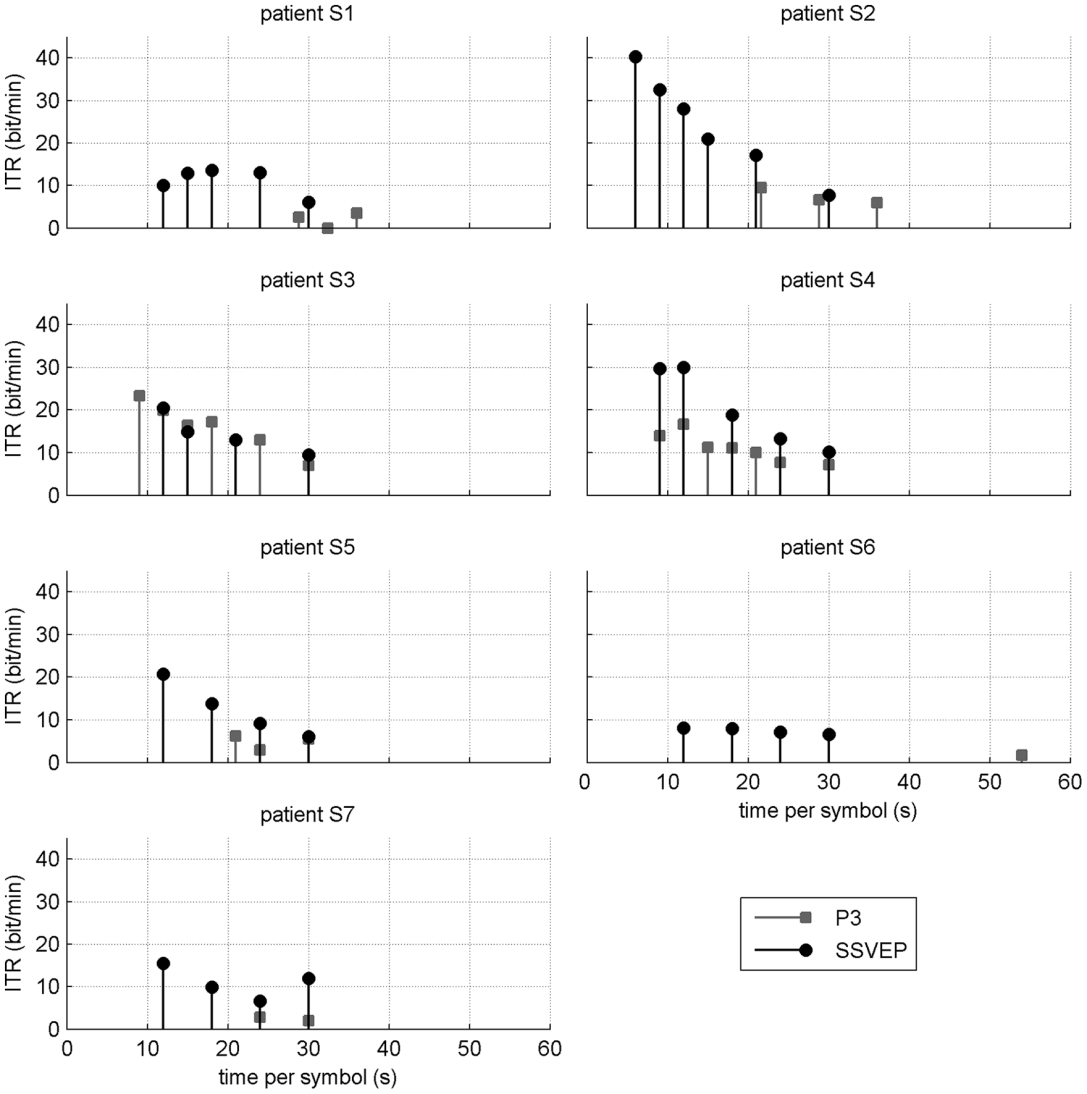
Information Transfer Rate (ITR) values for both BCIs and each patient with respect to the stimulation time per symbol. Note that for all subjects except S3, ITR could get from twice up to four times higher for the SSVEP-based BCI than for the P3-based one.

The corresponding ITR values are shown in Fig. 6. The highest value for the SSVEP-based BCI was 40 bit/min (patient S2), while the highest value for the P3-based BCI was 23 bit/min (patient S3). For a given participant (except S3), the best ITR value could get from twice up to four times higher for the SSVEP- than for the P3-based BCI.

**Figure 7:**
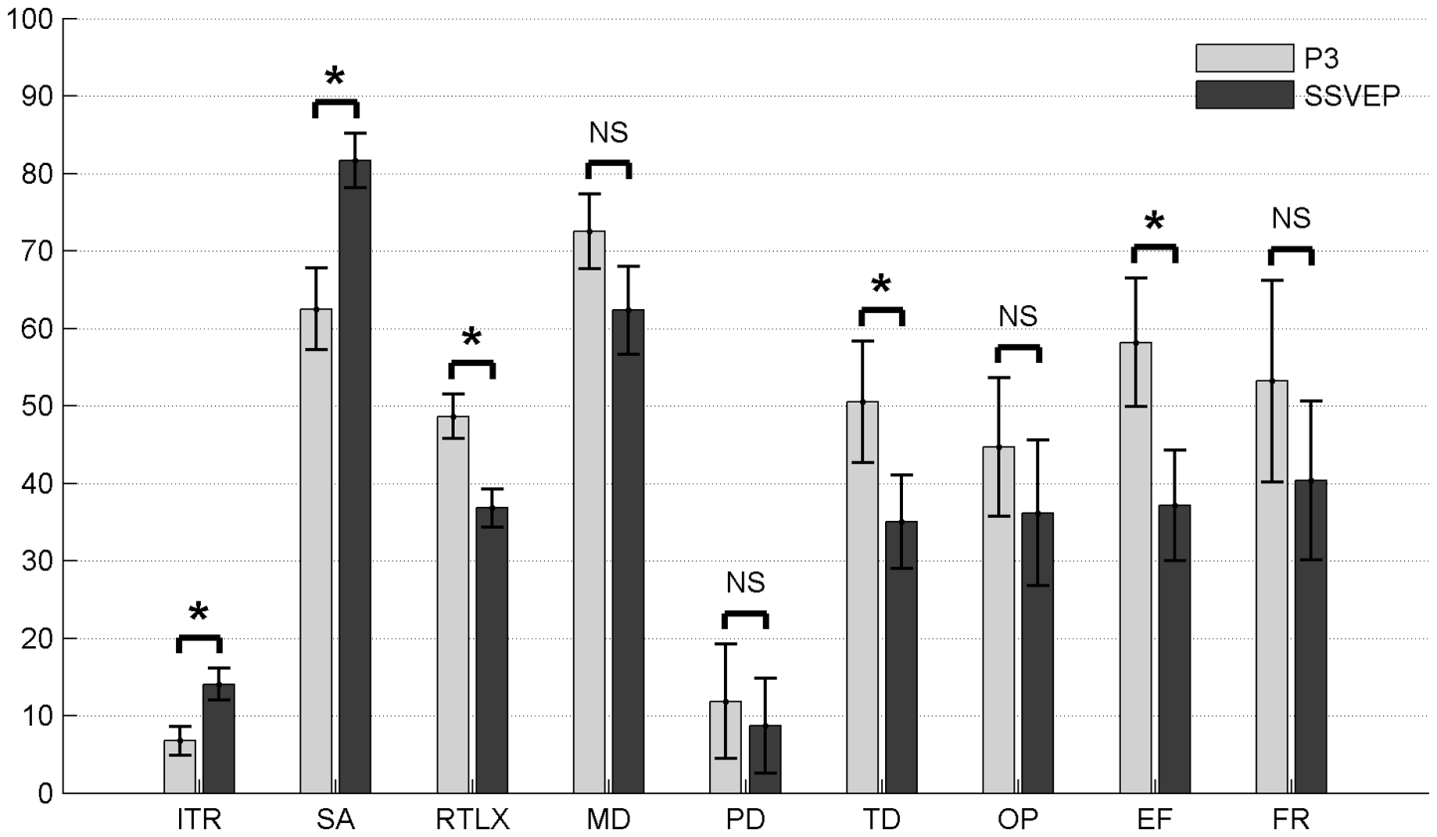
Boxplots of the values for the Information Transfer Rate (ITR, bit/min) and the subjects' rating of the system (values ranging from 0 to 100) including satisfaction (SA), raw TLX (RTLX) and the 6 components of the NASA-TLX: mental demand (MD), physical demand (PD), temporal demand (TD), own performance (OP), effort (EF) and frustration (FR) for both BCI systems. An asterisk (*) denotes statistical significance at 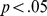 and NS stands for *Non-Significant* (Wilcoxon signed-rank test).

Global ITR values for each system and all patients are shown in Table 4. We observed for all of them, except S3, higher values for the SSVEP-based BCI. When averaging over participants (excluding S6), the ITR remained twice higher for the SSVEP-based BCI than for the P3-based one (see Fig. 7). On average, participants showed a significantly higher ITR with the SSVEP system than with the P3 one (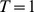, 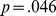).

**Table 4:** Patients' global Information Transfer Rate values (ITR), their responses to the NASA-TLX questionnaire, associated Raw TLX (RTLX) values, satisfaction and quality of life values for each BCI.

BCI	Patient	ITR	MD	PD	TP	OP	EF	FR	RTLX	SA	QoL
Xhline	S1	2.74	85	0	10	60	50	0	35	65	−2
	S2	6.87	85	0	50	10	60	60	44.17	80	3.5
	S3	15.22	55	0	60	55	80	85	56	50	3
P3	S4	9.37	80	10	60	20	80	60	51.67	779	2
	S5	4.17	70	51	53	53	59	24	51.67	54.7	−0.8
	S6	1.64	80	70	70	70	60	80	71.67	45.3	2
	S7	2.09	60	10	70	70	20	90	53.33	47.4	0
	S1	10.72	40	0	5	70	50	0	28.5	80	4
	S2	22.94	75	0	45	5	50	55	38.5	88	3.5
	S3	13.53	60	0	50	50	60	65	47.5	85	3
SSVEP	S4	18.19	50	5	40	10	30	70	34.17	88.4	2
	S5	9.62	79	42	40	32	23	22	39.67	63.2	−0.8
	S6	7.35	50	70	70	30	30	80	55	84.2	2
	S7	9.33	70	5	30	50	10	30	32.5	85.3	0

The RTLX is the mean of the values given by the patients to each of the 6 subscales of the NASA-TLX questionnaire (all values ranging from 0 to 100): mental demand (MD), physical demand (PD), temporal demand (TD), own performance (OP), effort (EF) and frustration (FR). SA denotes the satisfaction index (ranging from 0 to 100) and QoL the quality of life index (ranging from −5 to +5).

### Mental workload and satisfaction comparison

The patients' responses to the satisfaction, ACSA, and NASA-TLX questionnaires are shown in Table 4 for both BCIs. This table also shows the estimated mental workload (RTLX) calculated from the patients' ratings of the 6 scales defined in the NASA-TLX. The averaged values over participants (excluding S6) are shown in Fig. 7.

For each participant the RTLX value was higher for the P3- than for the SSVEP-based system. On average, the P3-based BCI led to higher mental workload than the SSVEP-based BCI (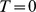, 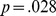). Among the factors characterizing the mental workload, the ones for which the P3-based BCI led to significantly higher values were the temporal demand (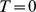, 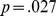) and the effort (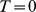, 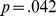).

Similarly, all patients were more satisfied with the SSVEP-based system than with the P3-based one. Statistically they were on average more satisfied with the SSVEP-based BCI (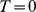, 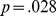).

### Level of impairment, quality of life and performance

The severity of motor impairments measured by the Patterson and the ALSFRS-R scales are reported in Table 1.

No significant correlation between the ITR and the Patterson scores was found for our population of patients (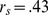, 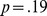). When looking separately at each BCI, no significant correlation was found neither for the P3-based one (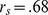, 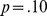), nor for the SSVEP-based one (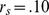, 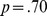).

**Figure 8:**
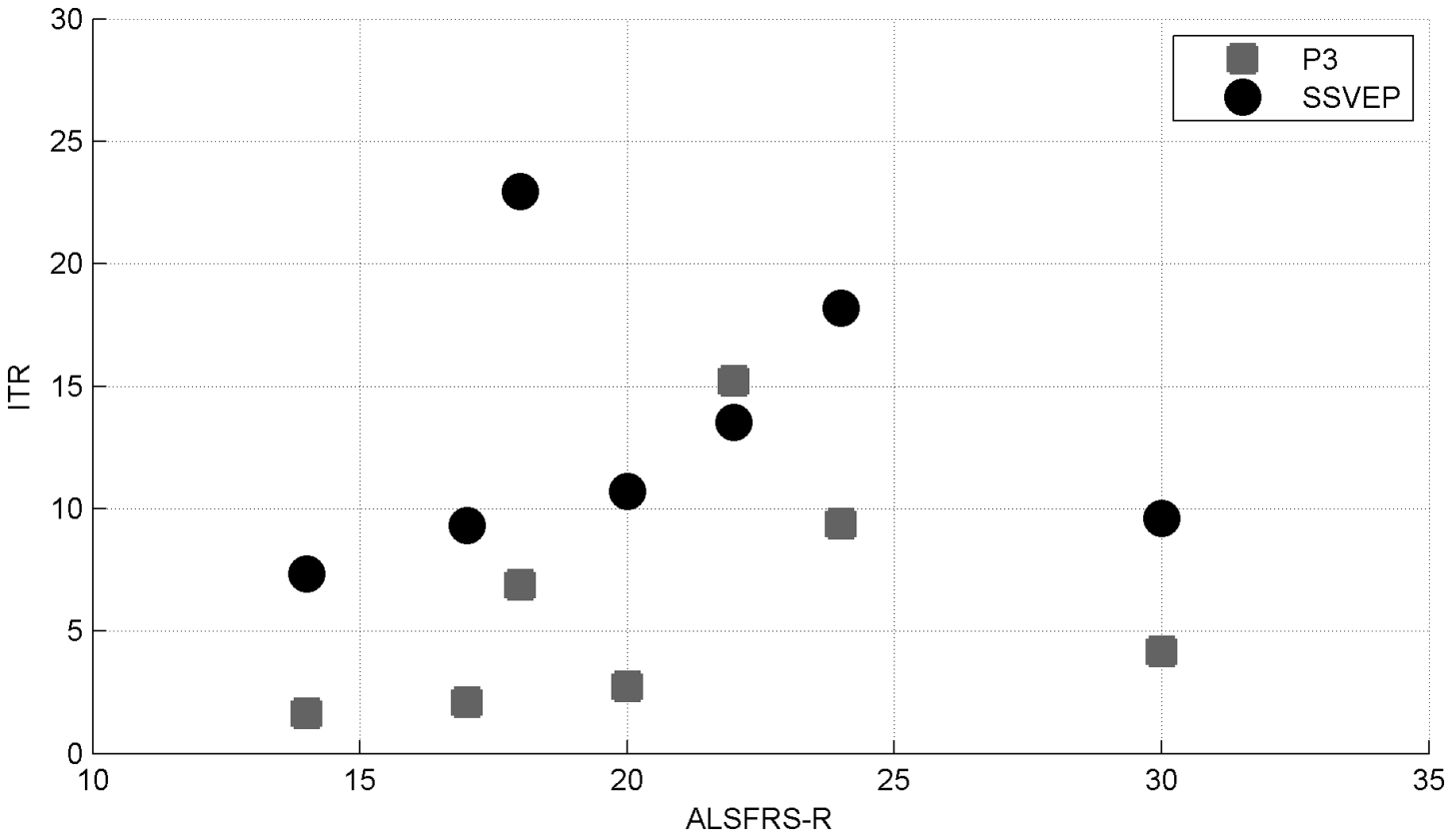
Information Transfer Rate for all patients and for both systems plotted against the patients' ALSFRS-R scores. Note that the two most impaired patients have the lowest performance for both BCIs; however, no correlation was found between those two measures.

Fig. 8 displays for both BCI systems and for each patient the ITR plotted against the ALSFRS-R score. It shows that the 2 patients with the lowest ALSFRS-R score (S6 and S7) are the ones with the lowest performance for both systems. On the other hand, it is also clearly visible that the least impaired patient of our population (S5) is also among the participants with the lowest performance.

No significant correlation between the ITR and the ALSFRS-R scores was found for our population of patients (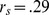, 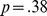). When looking separately at each BCI, no significant correlation was found neither for the P3-based one (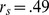, 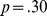), nor for the SSVEP-based one (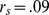, 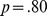). The quality of life of the patients was neither related to their level of impairment (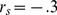, 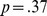), nor to their BCI performance (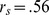, 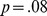).

## Discussion

We here assessed a P3- and an SSVEP-based spelling application on a cohort of incomplete LIS patients. In order to be applicable for daily use, these BCI systems need not only to be accurate, but also to be easy to use for the patient. Their applicability therefore depends not only on the achieved performance but also on the users' assessment of the mental workload associated with the BCI task and the overall satisfaction with the BCI system. For this reason, the comparison was done in terms of typing performance, mental workload and user satisfaction.

We found that all 7 patients were able to overcome the usability threshold of 70% for typing accuracy with the SSVEP-based BCI, while this was the case for only 3 of them (S2, S3 and S4) with the P3-based BCI. Moreover, for 2 patients (S6 and S7) the accuracy was below the 50% threshold for the P3 system, meaning that for them this system was not usable. This seems to contradict the results obtained by [27] who observed that more subjects were able to use a P3-based BCI than a SSVEP-based one; however, they considered healthy participants only. This supports the notion that results obtained with a healthy group are not always generalizable to a patient group [29].

Moreover, we observed a significantly better performance with the SSVEP-based system than with the P3-based one. More precisely, for 6 patients out of 7, the SSVEP-based BCI typically led to higher accuracies and faster settings could be applied for most of them. Only one patient (S3) achieved a slightly better performance with the P3-based BCI. Those results are also supported by the higher ITR values observed with the SSVEP-based application (also observed by [27]). Indeed, for a given participant (except S3), the best ITR value could get from twice up to four times higher for the SSVEP-based BCI than for the P3-based one.

For each participant, the subjective mental workload was higher with the P3- than with the SSVEP-based BCI. Similarly, and in agreement with the results from [27], all patients were more satisfied with the SSVEP-based BCI than with the P3-based one.

It is important to stress that we compared two systems based on classical paradigms (8 electrodes placed above the frontoparietal or occipital cortex; specific design for the stimuli presentation, signal processing and classification methods). However, additional recording sites [9], [10], [68], designs [41], [43], [44], [69], [70] and classifiers [14], [71] have been reported to influence performances achieved with those systems. Thus different results could be obtained using different electrode sites, stimulation design and/or signal processing/classification techniques.

Moreover, given the small number of participants and sessions, we do not claim that our conclusions on usability pertain to all LIS patients. Additionally, patients were given complete freedom regarding the words they chose to communicate and the speed settings of the BCIs (number of repetitions/stimulus duration). This approach has the advantage that the data were collected in a situation as close as possible to how they would use the system in a real daily use home setting. However it also means that the number of symbols communicated per subject, BCI system, session and speed setting were not constant (see Supplementary Materials S1 and S2 for details of each session) which limits the performance estimation and performed comparisons.

In summary, our study suggests that for the tested population of patients, the SSVEP-based BCI leads to a better usability than the P3-based BCI based on all the considered criteria. Indeed, the better performance of the SSVEP system was accompanied by a lower mental workload and a higher satisfaction for this population of patients. To our knowledge, this is the first study assessing both an SSVEP and a P3-based letter-spelling BCIs in the challenging setting of LIS.

### P3 and SSVEP responses

We can try to interpret our results by putting them alongside previous studies investigating the cognitive processes behind P3 and SSVEP responses. The P3 has been regarded as a cognitive potential depending on attention and working memory (for a review, see [72]). In support of this idea, [73] observed an increased latency and decreased amplitude of the P3 for an increased difficulty to discriminate the two stimuli of an oddball task (standard and target). Indeed, this response, appearing in the parietal cortex, seems to involve other cortical areas such as hippocampus and cingulate cortex suggesting a more complex processing of the stimulus. Moreover, it has also been shown that the P3 could be used as an indicator of attentional dysfunctions in pathological populations such as attention deficit hyperactivity disorder [74], mild cognitive impairment and dementia [75], [76].

According to previous studies, our results could suggest that the P3-based BCI is cognitively more demanding than the SSVEP-based one. Indeed, the P3 response seems to involve a sequential activation of cortical areas compared to SSVEP response which seems to rely less on cognitive abilities [34], [35], [77], [78].

Contrarily to our results, [27] found that it was more likely for healthy subjects to control a P3-based BCI than a SSVEP-based one and that both systems led to similar levels of accuracy. This difference in results could be explained by the conditions of our patients. Indeed, using a similar P3-based BCI as the one we used here, [30] observed in motor disabled participants a reduced cortical differentiation and specialization leading to the recruitment of more cortical areas to perform the spelling task and reflecting a less efficient operating strategy. They also observed a smaller ITR and accuracy in motor disabled as compared to healthy controls.

We hypothesize that the patients included in our study are more sensitive to a system that implies a more complex cognitive processing than healthy volunteers leading to a higher mental workload and an inferior performance and satisfaction. Results from the preliminary tests (see Fig. 1 and Fig. 2) showed for all patients a preserved oddball and SSVEP response similar to what has been observed in healthy controls (*e.g.* [21], [72]), suggesting a spared basic attentional capability in our population of patients (see also a study assessing cognitive functions in LIS patients with an adapted neuropsychological battery in [79]).

Both BCI systems tested here rely on gaze control and all the patients tested were able to direct their gaze toward their target on the screen. In both cases of P3- and SSVEP-based BCIs, there exist studies describing gaze independent alternatives (see [70], [80]–[82]), though performances are typically lower than with gaze dependent systems. To our knowledge such covert attention based BCIs have never been tested on locked-in patients, and as the results obtained in the present study cannot be generalized to the covert attention case, more studies are needed to investigate the efficiency and to compare designs and signal modality of systems using covert attention in this population.

### Level of impairment, quality of life and BCI performance

BCI technology aims at providing a communication channel to severely motor impaired patients. However this target population is very heterogeneous in terms of level of disability [3], and little is known about the eventual relation between the degree of impairment of a patient and the BCI performance.

As can be seen from Table 1, all patients had a score of 2 or 3 on the 5 points Patterson scale. This scale was not sensitive enough to reflect differences in the level of impairment between the patients participating in the study. For this reason, we used the ALSFRS-R scale, which was able to make a clearer distinction between the patients and allowed for a more qualitative study of the relationship between the level of impairment and the performance.

As observed by [16] and [17], we did not find any correlation between the level of impairment and the BCI performance. While this absence of correlation could be due the small amount of patients tested, we noticed however, that the 2 most impaired patients who participated in our study achieved the lowest performance with both systems. Additionally, results from other studies seem to indicate that the most disabled patients tend to perform the worst when using a BCI. For example, [19] observed a significantly worse performance for the 2 most impaired patients (out of 5 tetraplegic patients) compared to a control group composed of 7 healthy subjects when using a P3-based BCI. In their study, [53] found a significant correlation between physical impairment and BCI performance. However, this relationship disappeared when excluding patients in a complete LIS state. As the most severely impaired patients are the ones in the highest need of an alternative way of communication, it is important to include such patients when performing BCI studies and to develop systems that would give them a sufficient level of control for the system to be considered usable.

Looking at the effect of quality of life on performance and motor impairment, we did not find any correlation between the subjective assessment of the quality of life and the level of impairment or the BCI performance. Those results are in line with the ones observed by [58].

## Conclusion

Our study is the first assessing the applicability of two different BCIs in patients with incomplete LIS other than ALS. It is also the first one assessing an SSVEP- and a P3-based BCI on the same cohort of patients. The results show a better usability of the SSVEP-based BCI on the tested population of incomplete LIS patients based on performance (accuracy, speed, ITR), cognitive workload and user satisfaction criteria. Particularly, even the patients who had a level of control below the acceptable threshold (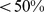 accuracy) when using the P3-based system were able to use the SSVEP-based system with an acceptable level of control (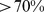 accuracy).

This study was designed to mimic as closely as possible a daily use of the system. Indeed, instead of looking for the best performance, the patient was always left with the choice to increase or decrease the stimulation duration according to his/her feeling of control over the BCI. Moreover, all tests were performed at the patient's home, which is a noisier environment than an in lab condition. Doing this, the performance, cognitive workload and satisfaction assessments provide a realistic impression of the applicability of the two systems in daily life.

However, those results are preliminary and need to be supported by results obtained in a broader population to be confirmed. Particularly considering the fact that the results obtained in this study could be due not only to the difference in signal modality (P3 *v.s.* SSVEP) but also to the differences in the design of each BCI. Future, more longitudinal studies are therefore needed to compare different type of P3- and SSVEP-based BCIs on a broader population of patients, including complete locked-in ones. If we assume, according to previous studies, that the performance obtained with a P3-based BCI should remain stable over time [16]–[18], it could be interesting to investigate a long term use of an SSVEP-based BCI in terms of performance, mental workload and user satisfaction.

## Supporting Information

Supplementary Materials S1 (PDF)

Supplementary Materials S2 (PDF)
