# Supplementary material for: A Comparison of Two Spelling Brain-Computer Interfaces Based on Visual P3 and SSVEP in Locked-In Syndrome
Source: PLoS One. 2013 Sep 25;8(9):e73691. doi: 10.1371/journal.pone.0073691 (PMC3783473; doi:10.1371/journal.pone.0073691)
Supplement: Supplementary Materials S1 — (PDF) [file pone.0073691.s001.pdf]

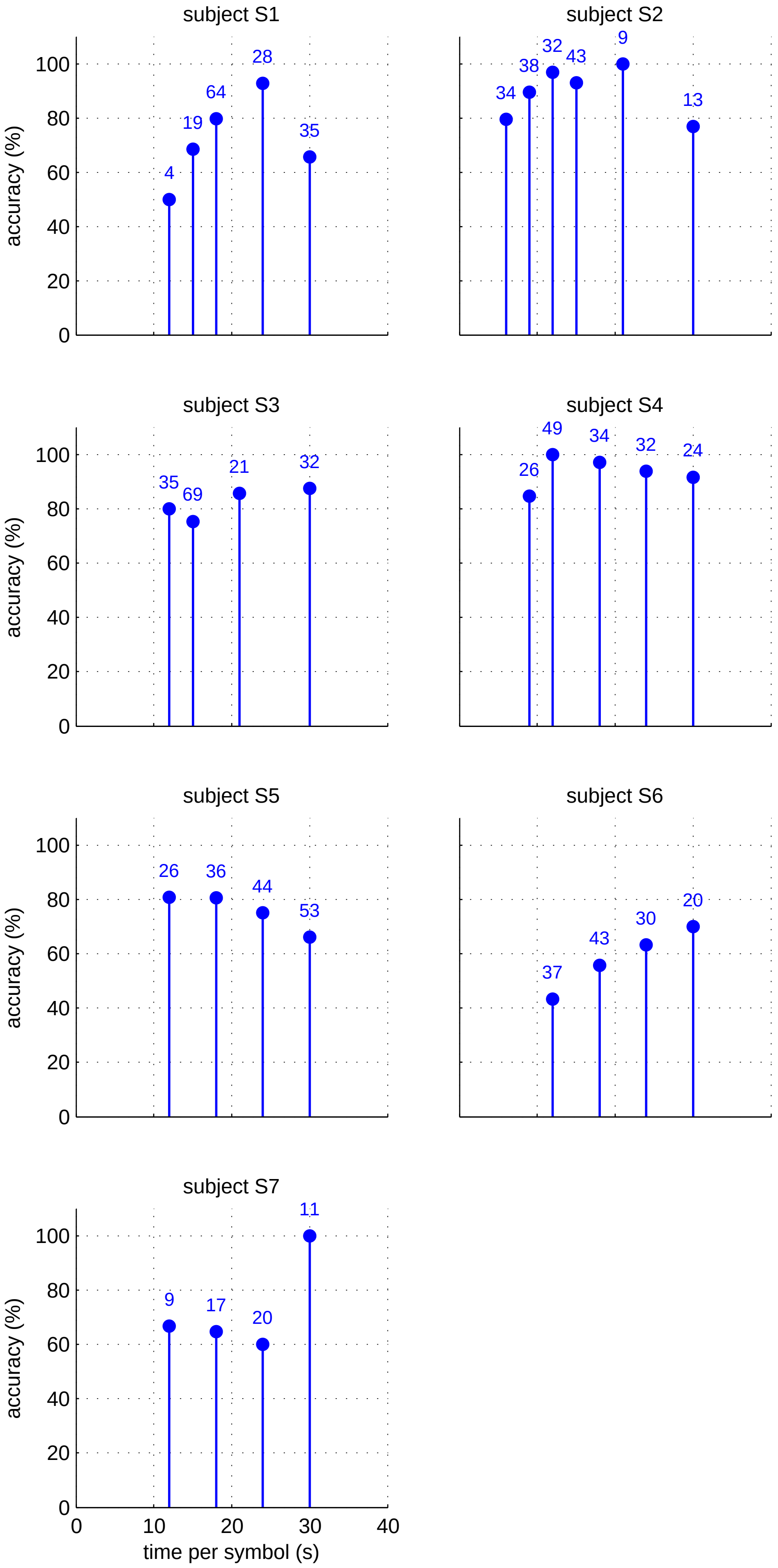

Figure 1. Detection accuracies for the SSVEP-based BCI and each patient with respect to the stimulation time per symbol. The figure above each data point represents the number of symbols communicated.

Table 1. detailed results for patient 1 per session using the SSVEP-based BCI. SD represents the stimulus duration, TST the total stimulation time in seconds corresponding to the communication of all symbol, MST the mean stimulation time in seconds corresponding to the communication of one symbol (average for the total line), CR the correctness ratio (number of correclty detected symbol *w.r.t.* total number of typed symbols) and the corresponding accuracy (%) and ITR the Information Transfer Rate.

SD	session 1				session 2				all sessions			
	**TST**	**MST**	**CR**	**ITR**	**TST**	**MST**	**CR**	**ITR**	**TST**	**MST**	**CR**	**ITR**
10	330	30	11/11 (100)	12	720	30	12/24 (50)	4.023	1050	30	23/35 (65.71)	6.046
8	480	24	20/20 (100)	15	192	24	6/8 (75)	9.236	672	24	26/28 (92.86)	13
6	432	18	21/24 (87.5)	15.7	720	18	30/40 (75)	12.31	1152	18	51/64 (79.69)	13.53
5	150	15	9/10 (90)	19.73	135	15	4/9 (44.44)	6.753	285	15	13/19 (68.42)	12.85
4	48	12	2/4 (50)	10.06	-	-	-	-	48	12	2/4 (50)	10.06
total	1440	20.87	63/69 (91.3)	14.53	1767	21.81	52/81 (64.2)	8.028	3207	21.38	115/150 (76.67)	10.72

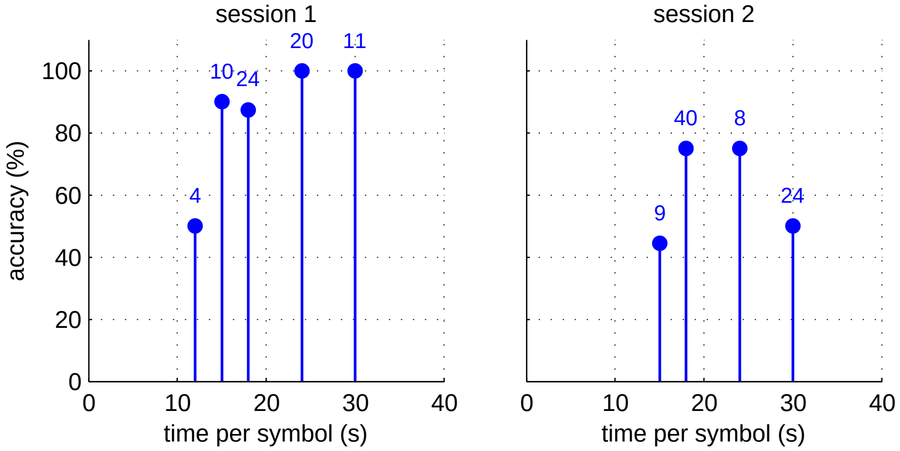

Figure 2. Detection accuracies for each session with patient 1 using the SSVEP-based BCI with respect to the stimulation time per symbol. The figure above each data point represents the number of symbols communicated.

Table 2. detailed results for patient 2 per session using the SSVEP-based BCI. SD represents the stimulus duration, TST the total stimulation time in seconds corresponding to the communication of all symbol, MST the mean stimulation time in seconds corresponding to the communication of one symbol (average for the total line), CR the correctness ratio (number of correclty detected symbol *w.r.t.* total number of typed symbols) and the corresponding accuracy (%) and ITR the Information Transfer Rate.

**SD**	**session 1**				**session 2**				**all sessions**			
	**TST**	**MST**	**CR**	**ITR**	**TST**	**MST**	**CR**	**ITR**	**TST**	**MST**	**CR**	**ITR**
10	120	30	4/4 (100)	12	270	30	6/9 (66.67)	6.179	390	30	10/13 (76.92)	7.683
7	189	21	9/9 (100)	17.14	-	-	-	-	189	21	9/9 (100)	17.14
5	135	15	9/9 (100)	24	510	15	31/34 (91.18)	20.17	645	15	40/43 (93.02)	20.87
4	84	12	7/7 (100)	30	300	12	24/25 (96)	27.59	384	12	31/32 (96.88)	28.06
3	162	9	16/18 (88.89)	32.22	180	9	18/20 (90)	32.89	342	9	34/38 (89.47)	32.57
2	132	6	19/22 (86.36)	46.1	72	6	8/12 (66.67)	30.89	204	6	27/34 (79.41)	40.36
total	822	11.91	64/69 (92.75)	26.15	1332	13.32	87/100 (87)	21.02	2154	12.75	151/169 (89.35)	22.94

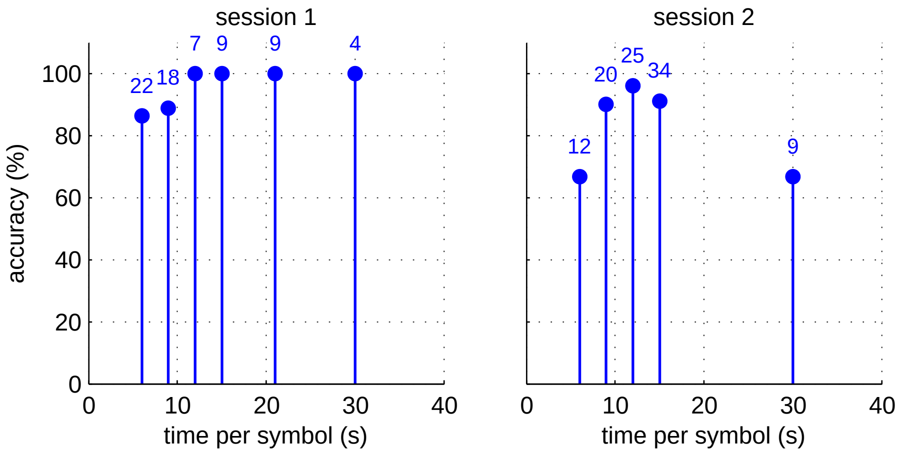

Figure 3. Detection accuracies for each session with patient 2 using the SSVEP-based BCI with respect to the stimulation time per symbol. The figure above each data point represents the number of symbols communicated.

Table 3. detailed results for patient 3 per session using the SSVEP-based BCI. SD represents the stimulus duration, TST the total stimulation time in seconds corresponding to the communication of all symbol, MST the mean stimulation time in seconds corresponding to the communication of one symbol (average for the total line), CR the correctness ratio (number of correclty detected symbol *w.r.t.* total number of typed symbols) and the corresponding accuracy (%) and ITR the Information Transfer Rate.

SD	session 1				session 2				all sessions			
	TST	MST	CR	ITR	TST	MST	CR	ITR	TST	MST	CR	ITR
10	510	30	15/17 (88.24)	9.548	450	30	13/15 (86.67)	9.273	960	30	28/32 (87.5)	9.419
7	168	21	8/8 (100)	17.14	273	21	10/13 (76.92)	10.98	441	21	18/21 (85.71)	13.01
5	330	15	20/22 (90.91)	20.07	705	15	32/47 (68.09)	12.76	1035	15	52/69 (75.36)	14.89
4	420	12	28/35 (80)	20.41	-	-	-	-	420	12	28/35 (80)	20.41
total	1428	17.41	71/82 (86.59)	15.95	1428	19.04	55/75 (73.33)	11.25	2856	18.19	126/157 (80.25)	13.53

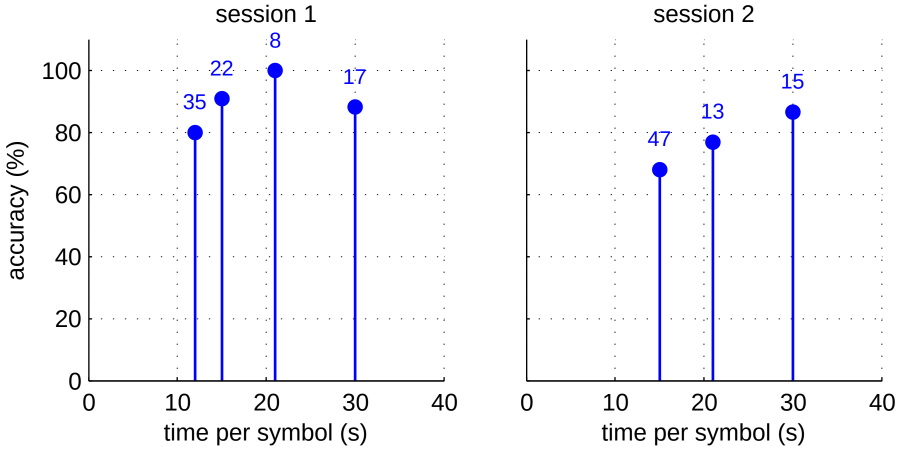

Figure 4. Detection accuracies for each session with patient 3 using the SSVEP-based BCI with respect to the stimulation time per symbol. The figure above each data point represents the number of symbols communicated.

Table 4. detailed results for patient 4 per session using the SSVEP-based BCI. SD represents the stimulus duration, TST the total stimulation time in seconds corresponding to the communication of all symbol, MST the mean stimulation time in seconds corresponding to the communication of one symbol (average for the total line), CR the correctness ratio (number of correclty detected symbol *w.r.t.* total number of typed symbols) and the corresponding accuracy (%) and ITR the Information Transfer Rate.

SD	session 1				session 2				all sessions			
	**TST**	**MST**	**CR**	**ITR**	**TST**	**MST**	**CR**	**ITR**	**TST**	**MST**	**CR**	**ITR**
10	300	30	10/10 (100)	12	420	30	12/14 (85.71)	9.109	720	30	22/24 (91.67)	10.18
8	432	24	18/18 (100)	15	336	24	12/14 (85.71)	11.39	768	24	30/32 (93.75)	13.22
6	288	18	16/16 (100)	20	324	18	17/18 (94.44)	17.86	612	18	33/34 (97.06)	18.78
4	276	12	23/23 (100)	30	312	12	26/26 (100)	30	588	12	49/49 (100)	30
3	-	-	-	-	234	9	22/26 (84.62)	29.74	234	9	22/26 (84.62)	29.74
total	1296	19.34	67/67 (100)	18.61	1626	16.59	89/98 (90.82)	18.11	2922	17.71	156/165 (94.55)	18.19

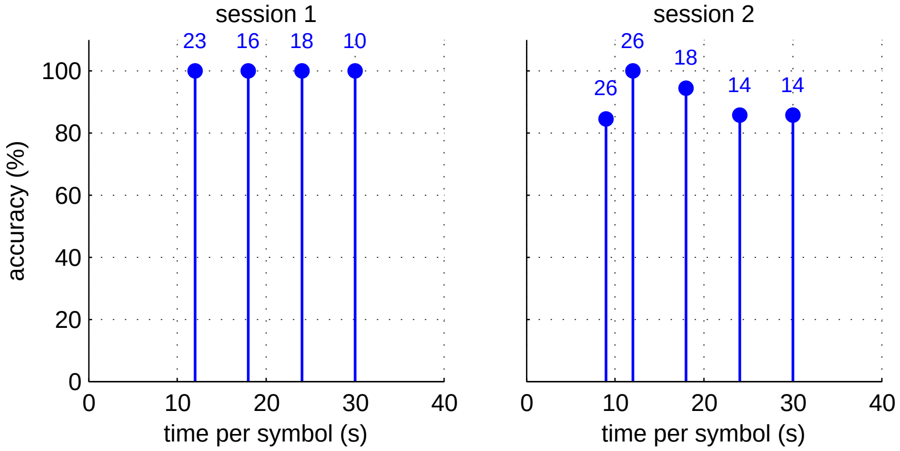

Figure 5. Detection accuracies for each session with patient 4 using the SSVEP-based BCI with respect to the stimulation time per symbol. The figure above each data point represents the number of symbols communicated.

Table 5. detailed results for patient 5 per session using the SSVEP-based BCI. SD represents the stimulus duration, TST the total stimulation time in seconds corresponding to the communication of all symbol, MST the mean stimulation time in seconds corresponding to the communication of one symbol (average for the total line), CR the correctness ratio (number of correclty detected symbol *w.r.t.* total number of typed symbols) and the corresponding accuracy (%) and ITR the Information Transfer Rate.

SD	session 1				session 2				all sessions			
	**TST**	**MST**	**CR**	**ITR**	**TST**	**MST**	**CR**	**ITR**	**TST**	**MST**	**CR**	**ITR**
10	690	30	18/23 (78.26)	7.89	900	30	17/30 (56.67)	4.845	1590	30	35/53 (66.04)	6.091
8	552	24	20/23 (86.96)	11.65	504	24	13/21 (61.9)	6.911	1056	24	33/44 (75)	9.236
6	432	18	22/24 (91.67)	16.96	216	18	7/12 (58.33)	8.432	648	18	29/36 (80.56)	13.76
4	312	12	21/26 (80.77)	20.72	-	-	-	-	312	12	21/26 (80.77)	20.72
total	1986	20.69	81/96 (84.38)	12.88	1620	25.71	37/63 (58.73)	5.962	3606	22.68	118/159 (74.21)	9.617

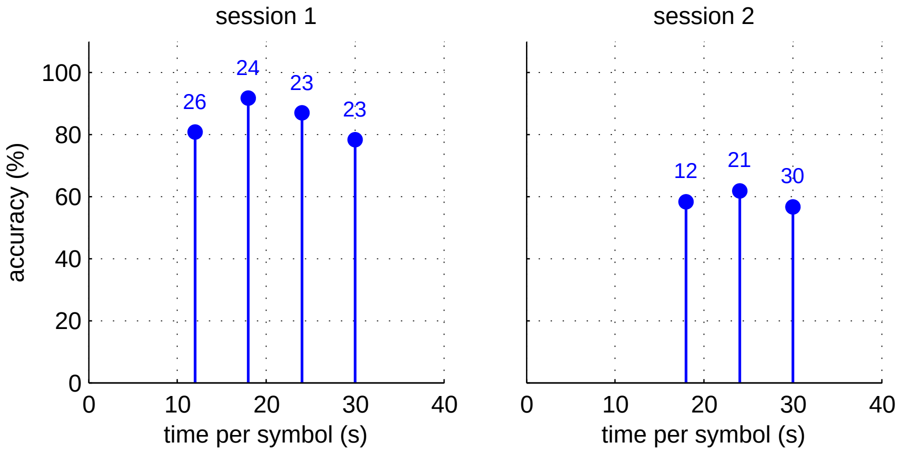

Figure 6. Detection accuracies for each session with patient 5 using the SSVEP-based BCI with respect to the stimulation time per symbol. The figure above each data point represents the number of symbols communicated.

Table 6. detailed results for patient 6 per session using the SSVEP-based BCI. SD represents the stimulus duration, TST the total stimulation time in seconds corresponding to the communication of all symbol, MST the mean stimulation time in seconds corresponding to the communication of one symbol (average for the total line), CR the correctness ratio (number of correclty detected symbol *w.r.t.* total number of typed symbols) and the corresponding accuracy (%) and ITR the Information Transfer Rate.

SD	session 1				session 2				all sessions			
	TST	MST	CR	ITR	TST	MST	CR	ITR	TST	MST	CR	ITR
10	300	30	8/10 (80)	8.165	300	30	6/10 (60)	5.276	600	30	14/20 (70)	6.651
8	456	24	13/19 (68.42)	8.032	264	24	6/11 (54.55)	5.723	720	24	19/30 (63.33)	7.151
6	234	18	8/13 (61.54)	9.133	540	18	16/30 (53.33)	7.379	774	18	24/43 (55.81)	7.896
4	132	12	6/11 (54.55)	11.45	312	12	10/26 (38.46)	6.802	444	12	16/37 (43.24)	8.104
total	1122	21.17	35/53 (66.04)	8.632	1416	18.39	38/77 (49.35)	6.436	2538	19.52	73/130 (56.15)	7.346

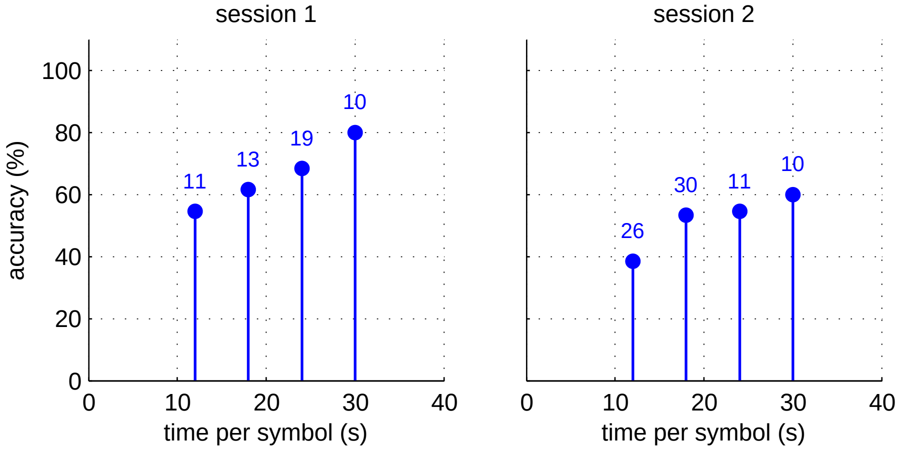

Figure 7. Detection accuracies for each session with patient 6 using the SSVEP-based BCI with respect to the stimulation time per symbol. The figure above each data point represents the number of symbols communicated.

Table 7. detailed results for patient 7 per session using the SSVEP-based BCI. SD represents the stimulus duration, TST the total stimulation time in seconds corresponding to the communication of all symbol, MST the mean stimulation time in seconds corresponding to the communication of one symbol (average for the total line), CR the correctness ratio (number of correclty detected symbol *w.r.t.* total number of typed symbols) and the corresponding accuracy (%) and ITR the Information Transfer Rate.

**SD**	**session 1**				**session 2**				**all sessions**			
	**TST**	**MST**	**CR**	**ITR**	**TST**	**MST**	**CR**	**ITR**	**TST**	**MST**	**CR**	**ITR**
10	180	30	6/6 (100)	12	150	30	5/5 (100)	12	330	30	11/11 (100)	12
8	312	24	7/13 (53.85)	5.614	168	24	5/7 (71.43)	8.573	480	24	12/20 (60)	6.595
6	198	18	5/11 (45.45)	5.819	108	18	6/6 (100)	20	306	18	11/17 (64.71)	9.846
4	-	-	-	-	108	12	6/9 (66.67)	15.45	108	12	6/9 (66.67)	15.45
total	690	23	18/30 (60)	6.882	534	19.78	22/27 (81.48)	12.75	1224	21.47	40/57 (70.18)	9.327

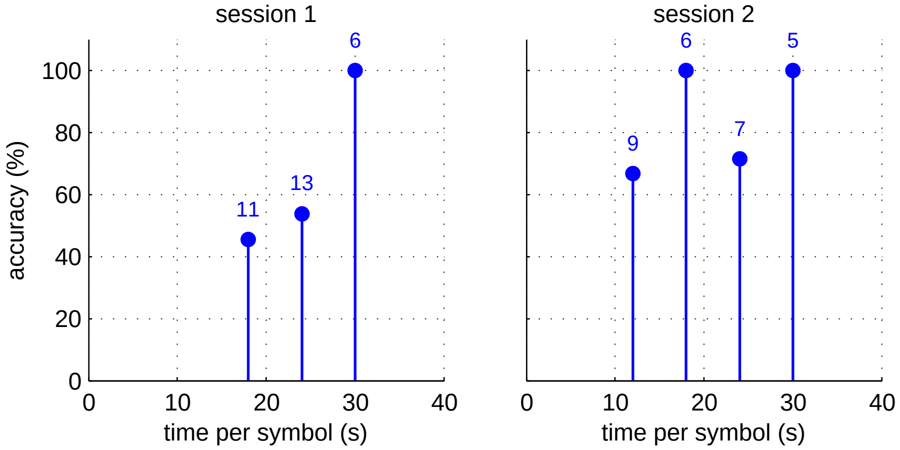

Figure 8. Detection accuracies for each session with patient 7 using the SSVEP-based BCI with respect to the stimulation time per symbol. The figure above each data point represents the number of symbols communicated.
