# Supplementary material for: A Comparison of Two Spelling Brain-Computer Interfaces Based on Visual P3 and SSVEP in Locked-In Syndrome
Source: PLoS One. 2013 Sep 25;8(9):e73691. doi: 10.1371/journal.pone.0073691 (PMC3783473; doi:10.1371/journal.pone.0073691)
Supplement: Supplementary Materials S2 — (PDF) [file pone.0073691.s002.pdf]

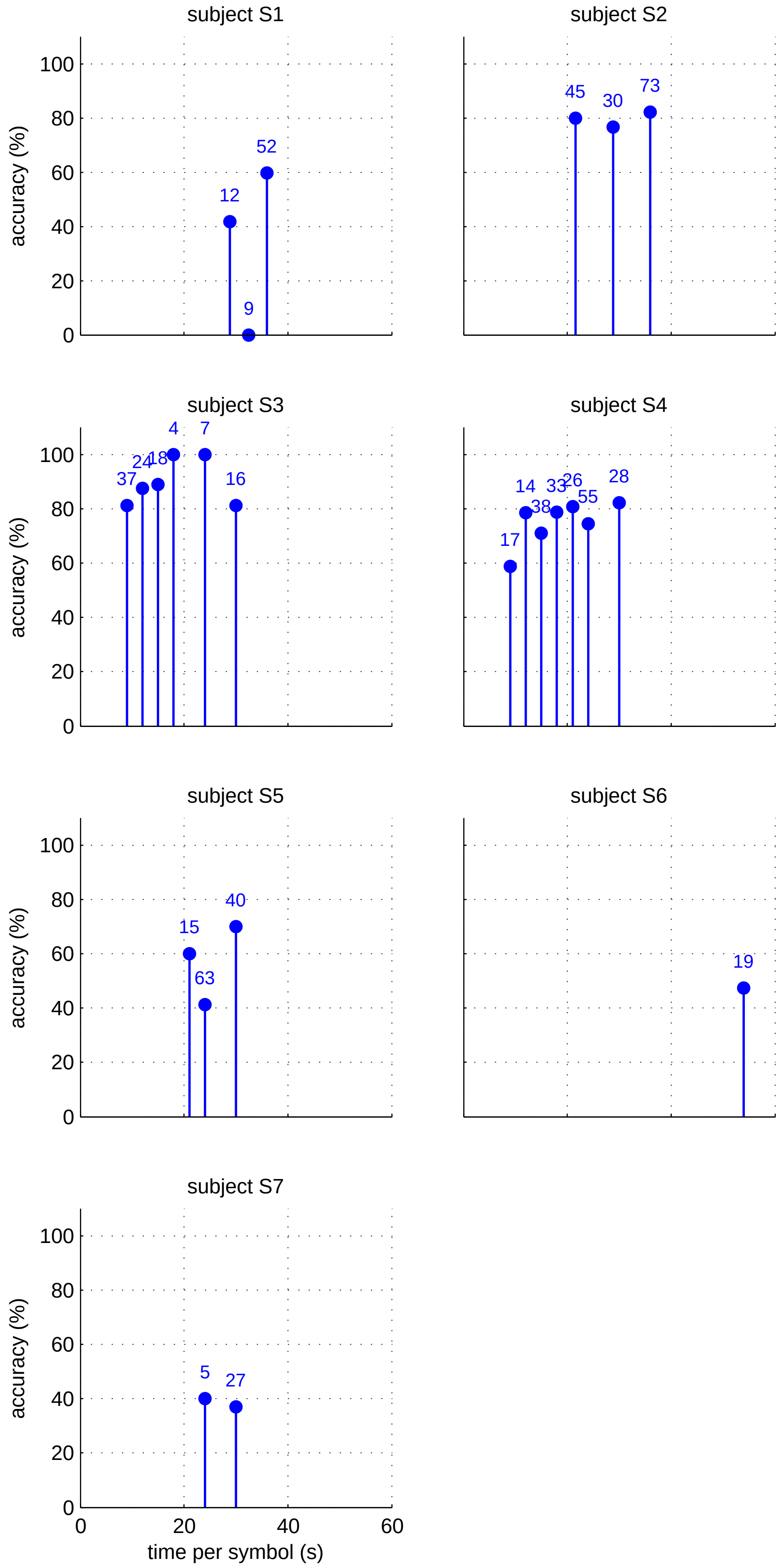

Figure 1. Detection accuracies for the P3-based BCI and each patient with respect to the stimulation time per symbol. The figure above each data point represents the number of symbols communicated.

Table 1. detailed results for patient 1 per session using the P3-based BCI. NR represents the number of repetitions, TST the total stimulation time in seconds corresponding to the communication of all symbol, SST the stimulation time in seconds corresponding to the communication of one symbol (average for the total line), CR the correctness ratio (number of correclty detected symbol *w.r.t.* total number of typed symbols) and the corresponding accuracy (%) and ITR the Information Transfer Rate.

NR	session 1				session 2				all sessions			
	TST	SST	CR	ITR	TST	SST	CR	ITR	TST	SST	CR	ITR
10	1188	36	18/33 (54.55)	3.074	684	36	13/19 (68.42)	4.417	1872	36	31/52 (59.62)	3.542
9	-	-	-	-	291.6	32.4	0/9 (0)	0	291.6	32.4	0/9 (0)	0
8	-	-	-	-	345.6	28.8	5/12 (41.67)	2.496	345.6	28.8	5/12 (41.67)	2.496
total	1188	36	18/33 (54.55)	3.074	1321	33.03	18/40 (45)	2.463	2509	34.37	36/73 (49.32)	2.741

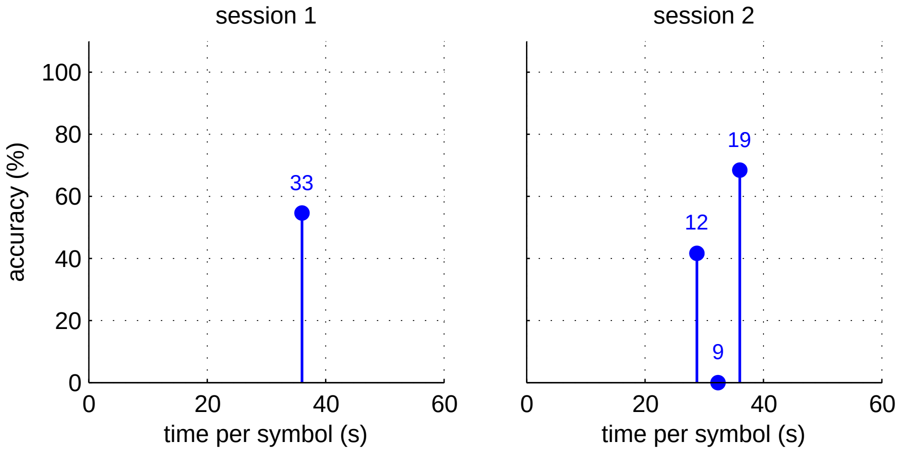

Figure 2. Detection accuracies for each session with patient 1 using the P3-based BCI with respect to the stimulation time per symbol. The figure above each data point represents the number of symbols communicated.

Table 2. detailed results for patient 2 per session using the P3-based BCI. NR represents the number of repetitions, TST the total stimulation time in seconds corresponding to the communication of all symbol, SST the stimulation time in seconds corresponding to the communication of one symbol (average for the total line), CR the correctness ratio (number of correclty detected symbol *w.r.t.* total number of typed symbols) and the corresponding accuracy (%) and ITR the Information Transfer Rate.

**NR**	**session 1**				**session 2**				**all sessions**			
	**TST**	**SST**	**CR**	**ITR**	**TST**	**SST**	**CR**	**ITR**	**TST**	**SST**	**CR**	**ITR**
10	1296	36	27/36 (75)	5.127	1332	36	33/37 (89.19)	6.869	2628	36	60/73 (82.19)	5.968
8	316.8	28.8	8/11 (72.73)	6.095	547.2	28.8	15/19 (78.95)	6.974	864	28.8	23/30 (76.67)	6.644
6	-	-	-	-	972	21.6	36/45 (80)	9.506	972	21.6	36/45 (80)	9.506
total	1613	34.31	35/47 (74.47)	5.317	2851	28.23	84/101 (83.17)	7.764	4464	30.16	119/148 (80.41)	6.865

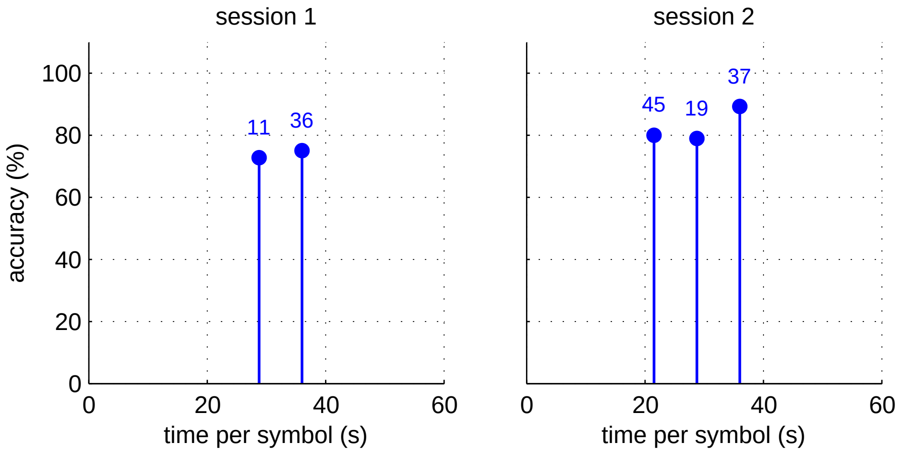

Figure 3. Detection accuracies for each session with patient 2 using the P3-based BCI with respect to the stimulation time per symbol. The figure above each data point represents the number of symbols communicated.

Table 3. detailed results for patient 3 per session using the P3-based BCI. NR represents the number of repetitions, TST the total stimulation time in seconds corresponding to the communication of all symbol, SST the stimulation time in seconds corresponding to the communication of one symbol (average for the total line), CR the correctness ratio (number of correclty detected symbol *w.r.t.* total number of typed symbols) and the corresponding accuracy (%) and ITR the Information Transfer Rate.

NR	session 1				session 2				all sessions			
	**TST**	**SST**	**CR**	**ITR**	**TST**	**SST**	**CR**	**ITR**	**TST**	**SST**	**CR**	**ITR**
10	210	30	6/7 (85.71)	7.691	270	30	7/9 (77.78)	6.532	480	30	13/16 (81.25)	7.024
8	168	24	7/7 (100)	12.92	-	-	-	-	168	24	7/7 (100)	12.92
6	72	18	4/4 (100)	17.23	-	-	-	-	72	18	4/4 (100)	17.23
5	-	-	-	-	270	15	16/18 (88.89)	16.39	270	15	16/18 (88.89)	16.39
4	192	12	13/16 (81.25)	17.56	96	12	8/8 (100)	25.85	288	12	21/24 (87.5)	19.93
3	-	-	-	-	333	9	30/37 (81.08)	23.33	333	9	30/37 (81.08)	23.33
total	642	18.88	30/34 (88.24)	12.85	969	13.46	61/72 (84.72)	16.81	1611	15.2	91/106 (85.85)	15.22

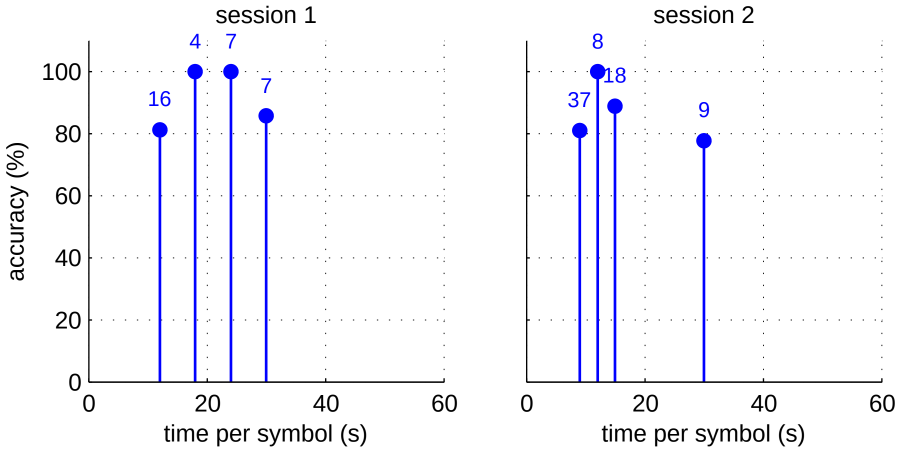

Figure 4. Detection accuracies for each session with patient 3 using the P3-based BCI with respect to the stimulation time per symbol. The figure above each data point represents the number of symbols communicated.

Table 4. detailed results for patient 4 per session using the P3-based BCI. NR represents the number of repetitions, TST the total stimulation time in seconds corresponding to the communication of all symbol, SST the stimulation time in seconds corresponding to the communication of one symbol (average for the total line), CR the correctness ratio (number of correclty detected symbol *w.r.t.* total number of typed symbols) and the corresponding accuracy (%) and ITR the Information Transfer Rate.

NR	session 1				session 2				all sessions			
	**TST**	**SST**	**CR**	**ITR**	**TST**	**SST**	**CR**	**ITR**	**TST**	**SST**	**CR**	**ITR**
10	450	30	12/15 (80)	6.844	390	30	11/13 (84.62)	7.523	840	30	23/28 (82.14)	7.154
8	648	24	19/27 (70.37)	6.934	672	24	22/28 (78.57)	8.303	1320	24	41/55 (74.55)	7.615
7	126	21	6/6 (100)	14.77	420	21	15/20 (75)	8.79	546	21	21/26 (80.77)	9.935
6	360	18	15/20 (75)	10.25	234	18	11/13 (84.62)	12.54	594	18	26/33 (78.79)	11.12
5	315	15	15/21 (71.43)	11.37	255	15	12/17 (70.59)	11.15	570	15	27/38 (71.05)	11.27
4	168	12	11/14 (78.57)	16.61	-	-	-	-	168	12	11/14 (78.57)	16.61
3	153	9	10/17 (58.82)	13.87	-	-	-	-	153	9	10/17 (58.82)	13.87
total	2220	18.5	88/120 (73.33)	9.618	1971	21.66	71/91 (78.02)	9.094	4191	19.86	159/211 (75.36)	9.365

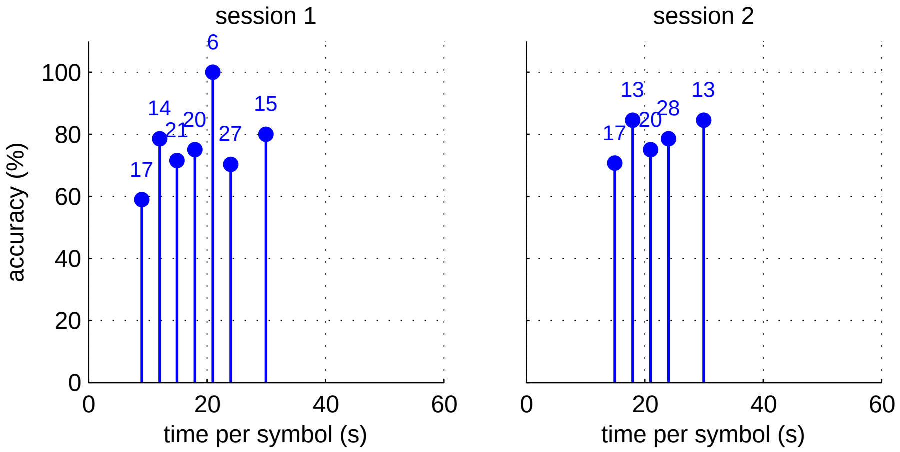

Figure 5. Detection accuracies for each session with patient 4 using the P3-based BCI with respect to the stimulation time per symbol. The figure above each data point represents the number of symbols communicated.

Table 5. detailed results for patient 5 per session using the P3-based BCI. NR represents the number of repetitions, TST the total stimulation time in seconds corresponding to the communication of all symbol, SST the stimulation time in seconds corresponding to the communication of one symbol (average for the total line), CR the correctness ratio (number of correclty detected symbol *w.r.t.* total number of typed symbols) and the corresponding accuracy (%) and ITR the Information Transfer Rate.

NR	session 1				session 2				all sessions			
	**TST**	**SST**	**CR**	**ITR**	**TST**	**SST**	**CR**	**ITR**	**TST**	**SST**	**CR**	**ITR**
10	660	30	16/22 (72.73)	5.851	540	30	12/18 (66.67)	5.084	1200	30	28/40 (70)	5.5
8	1056	24	18/44 (40.91)	2.907	456	24	8/19 (42.11)	3.046	1512	24	26/63 (41.27)	2.949
7	-	-	-	-	315	21	9/15 (60)	6.135	315	21	9/15 (60)	6.135
total	1716	26	34/66 (51.52)	3.885	1311	25.21	29/52 (55.77)	4.548	3027	25.65	63/118 (53.39)	4.169

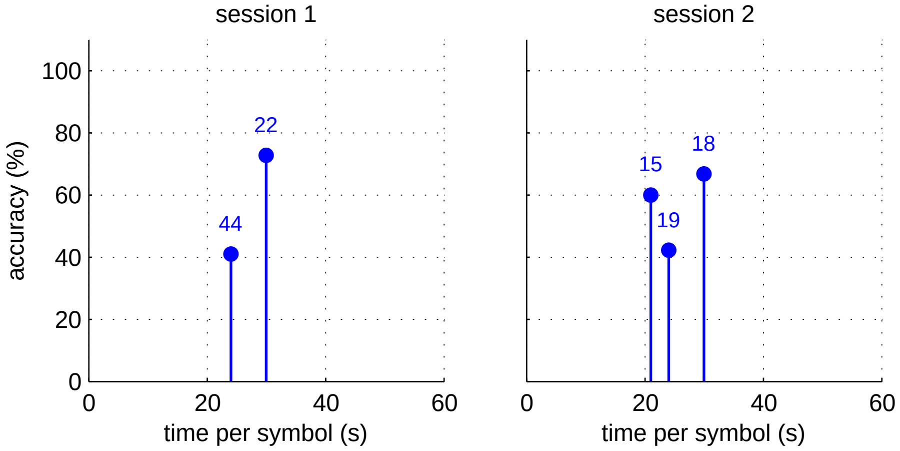

Figure 6. Detection accuracies for each session with patient 5 using the P3-based BCI with respect to the stimulation time per symbol. The figure above each data point represents the number of symbols communicated.

Table 6. detailed results for patient 6 per session using the P3-based BCI. NR represents the number of repetitions, TST the total stimulation time in seconds corresponding to the communication of all symbol, SST the stimulation time in seconds corresponding to the communication of one symbol (average for the total line), CR the correctness ratio (number of correclty detected symbol *w.r.t.* total number of typed symbols) and the corresponding accuracy (%) and ITR the Information Transfer Rate.

NR	session 1				all sessions			
	**TST**	**SST**	**CR**	**ITR**	**TST**	**SST**	**CR**	**ITR**
10	1026	54	9/19 (47.37)	1.636	1026	54	9/19 (47.37)	1.636
total	1026	54	9/19 (47.37)	1.636	1026	54	9/19 (47.37)	1.636

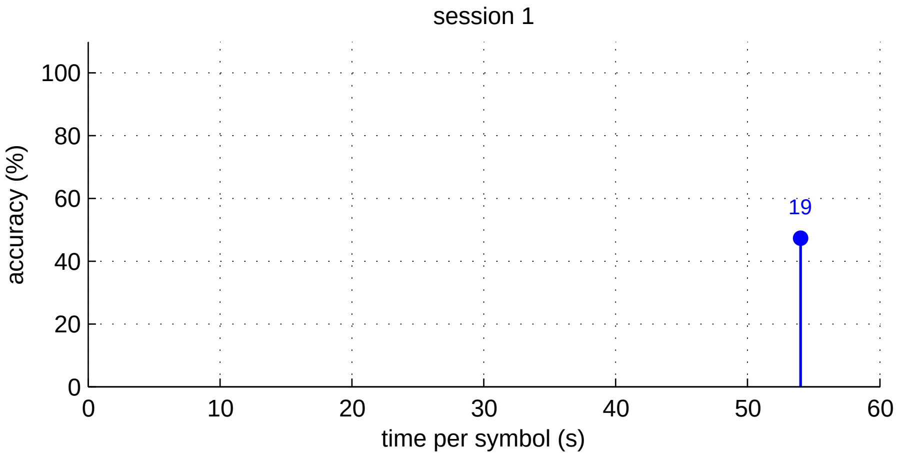

Figure 7. Detection accuracies for each session with patient 6 using the P3-based BCI with respect to the stimulation time per symbol. The figure above each data point represents the number of symbols communicated.

Table 7. detailed results for patient 7 per session using the P3-based BCI. NR represents the number of repetitions, TST the total stimulation time in seconds corresponding to the communication of all symbol, SST the stimulation time in seconds corresponding to the communication of one symbol (average for the total line), CR the correctness ratio (number of correclty detected symbol *w.r.t.* total number of typed symbols) and the corresponding accuracy (%) and ITR the Information Transfer Rate.

NR	session 1				session 2				all sessions			
	**TST**	**SST**	**CR**	**ITR**	**TST**	**SST**	**CR**	**ITR**	**TST**	**SST**	**CR**	**ITR**
10	360	30	8/12 (66.67)	5.084	450	30	2/15 (13.33)	0.3161	810	30	10/27 (37.04)	1.979
8	120	24	2/5 (40)	2.804	-	-	-	-	120	24	2/5 (40)	2.804
total	480	28.24	10/17 (58.82)	4.421	450	30	2/15 (13.33)	0.3161	930	29.06	12/32 (37.5)	2.085

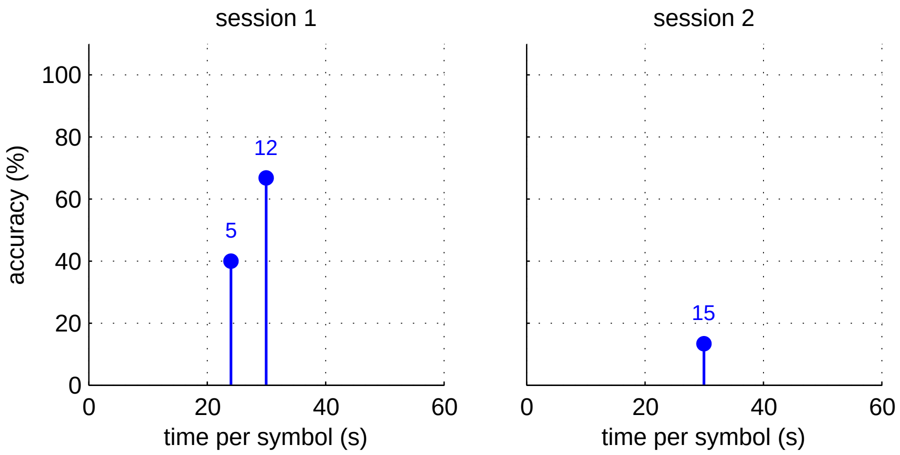

Figure 8. Detection accuracies for each session with patient 7 using the P3-based BCI with respect to the stimulation time per symbol. The figure above each data point represents the number of symbols communicated.
